# Macrophages and neutrophils are necessary for ER stress-induced β cell loss

**DOI:** 10.1016/j.celrep.2022.111255

**Published:** 2022-08-23

**Authors:** Bingyuan Yang, Liu Yang, Yueyang Wang, Lisette A. Maddison, Zihan Tang, Sander Haigh, Yulong Gong, Yue Zhang, Brittney A. Covington, Karin J. Bosma, Xin Tong, Patrick Page-McCaw, Maureen Gannon, Qing Deng, Wenbiao Chen

**Affiliations:** 1 Department of Molecular Physiology and Biophysics, Vanderbilt University School of Medicine, Nashville, TN 37232, USA; 2 Department of Endocrinology and Metabolism, Shanghai Tenth People’s Hospital, School of Medicine, Tongji University, No. 301 Middle Yanchang Road, Shanghai 200072, China; 3 Department of Biological Sciences, Purdue University, West Lafayette, IA 47907, USA; 4 Center for Reproductive Biology, Washington State University College of Veterinary Medicine, Pullman, WA 99164, USA; 5 State Key Laboratory of Freshwater Ecology and Biotechnology, Institute of Hydrobiology, Chinese Academy of Sciences, Wuhan, Hubei 430072, China; 6 Department of Veterans Affairs Tennessee Valley Authority, Nashville, TN, USA; 7 Department of Medicine, Vanderbilt University Medical Center, Nashville, TN, USA; 8 Lead contact

## Abstract

Persistent endoplasmic reticulum (ER) stress induces islet inflammation and β cell loss. How islet inflammation contributes to β cell loss remains uncertain. We have reported previously that chronic overnutrition-induced ER stress in β cells causes Ripk3-mediated islet inflammation, macrophage recruitment, and a reduction of β cell numbers in a zebrafish model. We show here that β cell loss results from the intricate communications among β cells, macrophages, and neutrophils. Macrophage-derived Tnfa induces *cxcl8a* in β cells. Cxcl8a, in turn, attracts neutrophils to macrophage-contacted “hotspots” where β cell loss occurs. We also show potentiation of chemokine expression in stressed mammalian β cells by macrophage-derived TNFA. In Akita and db/db mice, there is an increase in CXCL15-positive β cells and intra-islet neutrophils. Blocking neutrophil recruitment in Akita mice preserves β cell mass and slows diabetes progression. These results reveal an important role of neutrophils in persistent ER stress-induced β cell loss.

## In brief

Yang et al. show a pivotal role of communications among β cells, macrophages, and neutrophils in chronic overnutrition-induced loss of pancreatic β cells in a diabetes-prone zebrafish model.

## Graphical Abstract

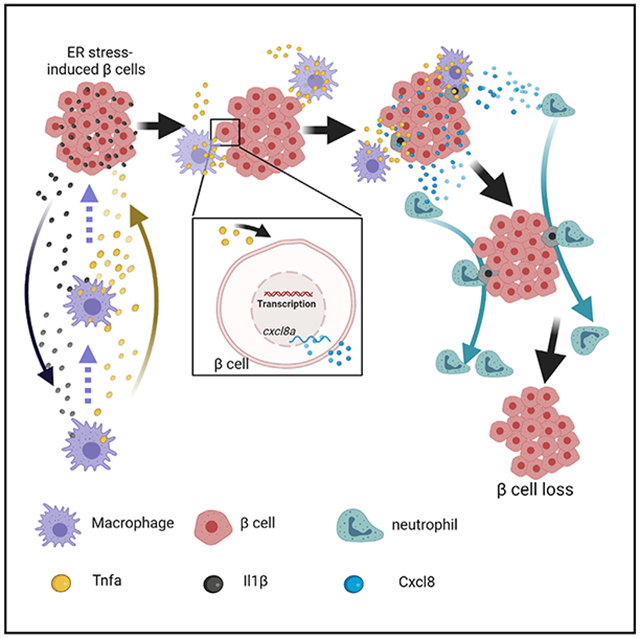

## INTRODUCTION

The prevalence of type 2 diabetes (T2D) in the United States has increased drastically in the past 4 decades because of obesity from overnutrition and sedentariness. Obesity creates the condition of insulin resistance (Qatanani and Lazar, 2007), which increases the workload of insulin-secreting β cells in pancreatic islets. Chronic stress causes β cell dysfunction and loss, resulting in insufficient insulin production (Halban et al., 2014; Weir et al., 2020). β cell reduction may result from a combination of cell death and loss of identity (Butler et al., 2003; Spijker et al., 2015; Talchai et al., 2012). Increased β cell death has been reported in several animal models of T2D (Dalbøge et al., 2013; Pick et al., 1998; Puff et al., 2011). The mechanisms by which chronic stress causes β cell death are a subject of active investigation.

ER stress from increased workload has been implicated in β cell death (Fonseca et al., 2011). The endoplasmic reticulum (ER) is essential for insulin biogenesis and secretion. A number of monogenic forms of diabetes have been linked to ER stress (Shrestha et al., 2021; Yong et al., 2021). Although prolonged ER stress can cause β cell death *in vitro* (Fonseca et al., 2011), ER stress rarely reaches such intensity *in vivo* (Shrestha et al., 2021). For example, Akita (Ins2^C96Y^) mice, a model of permanent neonatal diabetes because of accumulation of unfoldable insulin proteins in the ER, have decreased β cell mass primarily because of decreased postnatal expansion (Riahi et al., 2018). Increased β cell apoptosis in Akita mice has also been reported (Izumi et al., 2003; Oyadomari et al., 2002).

Islet inflammation has been implicated in T2D pathogenesis. In T2D, islet inflammation is mild and features a small increase in macrophages (Böni-Schnetzler and Meier, 2019; Butcher et al., 2014; Eguchi and Nagai, 2017; Ehses et al., 2007; Richardson et al., 2009). Whether the increase in islet macrophages is a cause or consequence of β cell death remains unresolved (Halban et al., 2014). In some T2D models, such as Goto-Kakizaki (GK) rats (Movassat et al., 1995) and fat sand rats (Jörns et al., 2006), increased islet macrophages are thought to be a consequence rather than a cause of β cell death (Cnop et al., 2005). In contrast, islet macrophages are necessary for β cell death in Zucker diabetic fatty (ZDF) rats (Jourdan et al., 2013).

Relative to the role of macrophages in β cell loss, much less is known about the role of neutrophils in β cell loss during the pathogenesis of T2D and other forms of non-autoimmune diabetes. Expression of CXCL8, a potent neutrophil chemokine, is increased in laser-dissected T2D β cells (Böni-Schnetzler et al., 2008; Igoillo-Esteve et al., 2010). The number of neutrophils, however, is not increased in T2D islets (Ehses et al., 2007). This may be due to the dynamic nature of neutrophils; they have a much shorter lifespan and much faster motility than macrophages, making it more difficult to detect changes in fixed tissue. It may also be due to the stage of disease progression in the donors. Live imaging may be necessary to determine whether neutrophils contribute to β cell loss.

We have reported previously that islet inflammation plays a critical role in persistent ER stress-induced β cell loss in a zebrafish model (Maddison et al., 2015; Yang et al., 2020). Using live imaging in the context of pharmacological and genetic manipulations, here we show that β cell loss involves intricate communications among β cells, macrophages, and neutrophils. Macrophages cause Tnfa-dependent induction of *cxcl8a* expression in β cells. Cxcl8a subsequently recruits neutrophils, which attack macrophage-contacted β cells and cause their loss. We also investigated whether these mechanisms are conserved in mammalian T2D model systems. We found that macrophage-derived TNFA induces *CXCL8* expression in human β cells or *Cxcl8* functional equivalents in mouse β cells. Islet neutrophils are increased in two mouse models of β cell loss involving persistent ER stress, db/db and Akita, and blocking neutrophil chemotaxis in Akita mice preserves β cell mass and slows diabetes progression. These studies reveal a previously unknown function of neutrophils in persistent ER stress-induced β cell loss.

## RESULTS

### Macrophages are necessary for β cell loss in the setting of overnutrition

**Figure 1.**
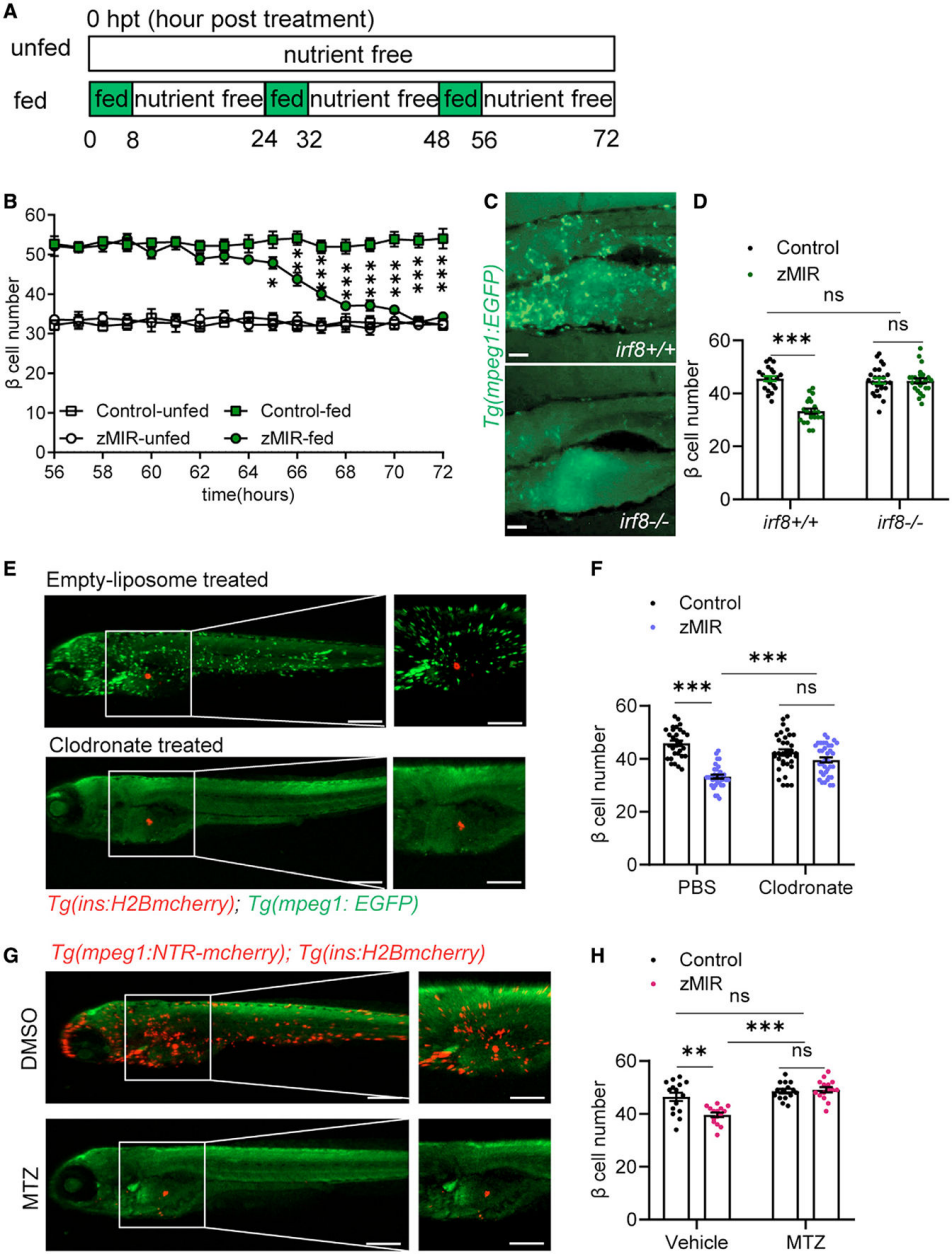
Macrophages are necessary for β cell loss in the setting of overnutrition (A) Schematic of the feeding regimen. Each day, fish were cultured for 8 h in nutrient-rich medium (5% chicken egg yolk) and for 16 h in nutrient-free medium, as indicated by the green and white rectangles, respectively. The 8-h culture in nutrient-rich medium is referred to as an overnutrition session. Time 0 starts at the onset of the first overnutrition 6 days post fertilization. (B) Detailed β-cell number dynamics during the 16 h of nutrient-free medium culture from 56–72 h (n > 15/time point). Data are mean ± SEM. Multiple t tests were performed within the fed and unfed groups. *p < 0.05, **p < 0.01, ***p < 0.001. (C) Macrophages in *irf8*^*−/−*^ fish (bottom) and control (top). Scale bars, 200 μm. (D) Effect of genetic deficiency of macrophages (*irf8*^*−/−*^) on β cell number. Data are mean ± SEM. n > 15/group, two-way ANOVA followed by Tukey’s multiple comparisons test; ns, not significant; ***p < 0.001. (E) Effect of clodronate liposomes (bottom) and empty liposomes (top) on macrophages. A green signal from *Tg(mpeg1:EGFP)* marks macrophages. A red signal from *Tg(ins:H2B-mcherry)* labels β cells. Liposomes were injected at 32 hpt, and fish were imaged at 64 hpt. Scale bars, 300 μm; scale bars within panels, 50 μm. See also Figure S1D. (F) Effect of clodronate liposomes on β cell loss in zMIR fish. Data are mean ± SEM. n > 20/group, two-way ANOVA followed by Tukey’s multiple comparisons test; ***p < 0.001. (G) Effect of metronidazole (MTZ) and DMSO on macrophages in zMIR fish with *Tg(mpeg1:NTR-mcherry)*. Scale bars, 300 μm; scale bars within panels, 50 μm. See also Figure S1F. (H) Effect of macrophage ablation by MTZ on β cell loss. Data are mean ± SEM. n > 15/group, two-way ANOVA followed by Tukey’s multiple comparisons test; **p < 0.01, ***p < 0.001.

We have reported previously that overnutrition induces β cell loss in zebrafish muscle insulin-resistant (zMIR) fish (Yang et al., 2020). The zMIR fish expresses dominant-negative Igf1r in skeletal muscle, which causes insulin resistance (Maddison et al., 2015). Overnutrition was achieved by culturing larvae in a 5% chicken egg yolk emulsion for 8 h during the daytime (Maddison and Chen, 2012), followed by 16 h in nutrient-free medium. After 3 consecutive days of overnutrition (Figure 1A), zMIR fish exhibit more severe ER stress in β cells, triggering Ripk3-mediated induction of *il1b* expression and subsequent macrophage recruitment, β cell loss, and glucose dyshomeostasis (Yang et al., 2020). The current study aims to determine the molecular and cellular mechanisms of β cell loss in this model.

We first investigated the time course of β cell loss in more detail. We designated the beginning of the first overnutrition session at 6 day-post-fertilization (dpf) as 0 h post treatment (hpt). We determined the β cell number in the principal islet of each fish at hourly intervals starting at 56 hpt, when the animals finished the third session of overnutrition. No significant difference was found at 56 hpt between control and zMIR fish (Figure S1A). Compared with the control, β cell numbers in zMIR fish declined between 62 and 71 hpt, with the steepest decline occurring from 65–68 hpt (Figure 1B). Because of the sensitivity of β cell loss to long immobilization (>2 h) (Yang et al., 2020), we focused our live imaging studies on 66–68 hpt.

We have shown previously that macrophages are found in the principal islet at 66 hpt, concomitant with β cell loss (Yang et al., 2020). To determine whether macrophage recruitment is a cause or a consequence of β cell loss, we assessed the effect of macrophage deficiency on overnutrition-induced β cell loss. First we investigated overnutrition-induced β cell loss in macrophage-deficient *irf8*^*ST95*^ mutants (Shiau et al., 2015). Throughout this study, macrophages were fluorescently labeled by fluorescent protein expression driven by the regulatory sequence of macrophage expressed gene 1 (*mpeg1*) (Felix et al., 2011). We confirmed that the *irf8*^*−/−*^ larvae have a marked reduction in macrophages at the time of β cell loss (Figure 1C). Compared with the *irf8*^*+/+*^ control, these fish produced a similar number of β cells during development and showed similar levels of β cell neogenesis in response to one session of overnutrition (Maddison and Chen, 2012; Figure S1B). At 56 hpt, *irf8*^*−/−*^; zMIR fish had a similar number of β cells as *irf8*^*+/+*^; zMIR controls. Unlike the *irf8*^*+/+*^; zMIR controls, *irf8*^*−/−*^; zMIR fish showed no overnutrition-induced β cell loss at 72 hpt (Figure 1D). We also found that controls had elevated whole-body free glucose, whereas *irf8*^*−/−*^ animals had normal whole-body free glucose (Figure S1C). As an alternative approach, we selectively ablated macrophages using clodronate liposomes (Carrillo et al., 2016; Figures 1E and S1D). Ablation of macrophages also reduced β cell loss in zMIR fish with minimal effect on control siblings (Figure 1F).

As a final approach to determine the role of macrophages in β cell loss, we generated zMIR animals with transgenic expression of bacterial nitroreductase (NTR) specifically in the macrophage lineage (Felix et al., 2011; Nguyen-Chi et al., 2015). This line allows controlled elimination of macrophages in the presence of metronidazole (MTZ) (Curado et al., 2008; Pisharath et al., 2007). As expected, administration of MTZ depleted macrophages (Figures 1G, S1E, and S1F). MTZ treatment prevented a decline of β cell numbers (Figure 1H) and an elevation of whole-body free glucose (Figure S1G). Thus, macrophage depletion by three independent methods resulted in preservation of β cell numbers and glucose homeostasis, demonstrating that macrophages play a causal role in β cell loss after overnutrition. We next determined the mechanisms underlying macrophage-dependent β cell loss.

### Macrophage-derived Tnfa is necessary for β cell loss in zMIR fish

**Figure 2.**
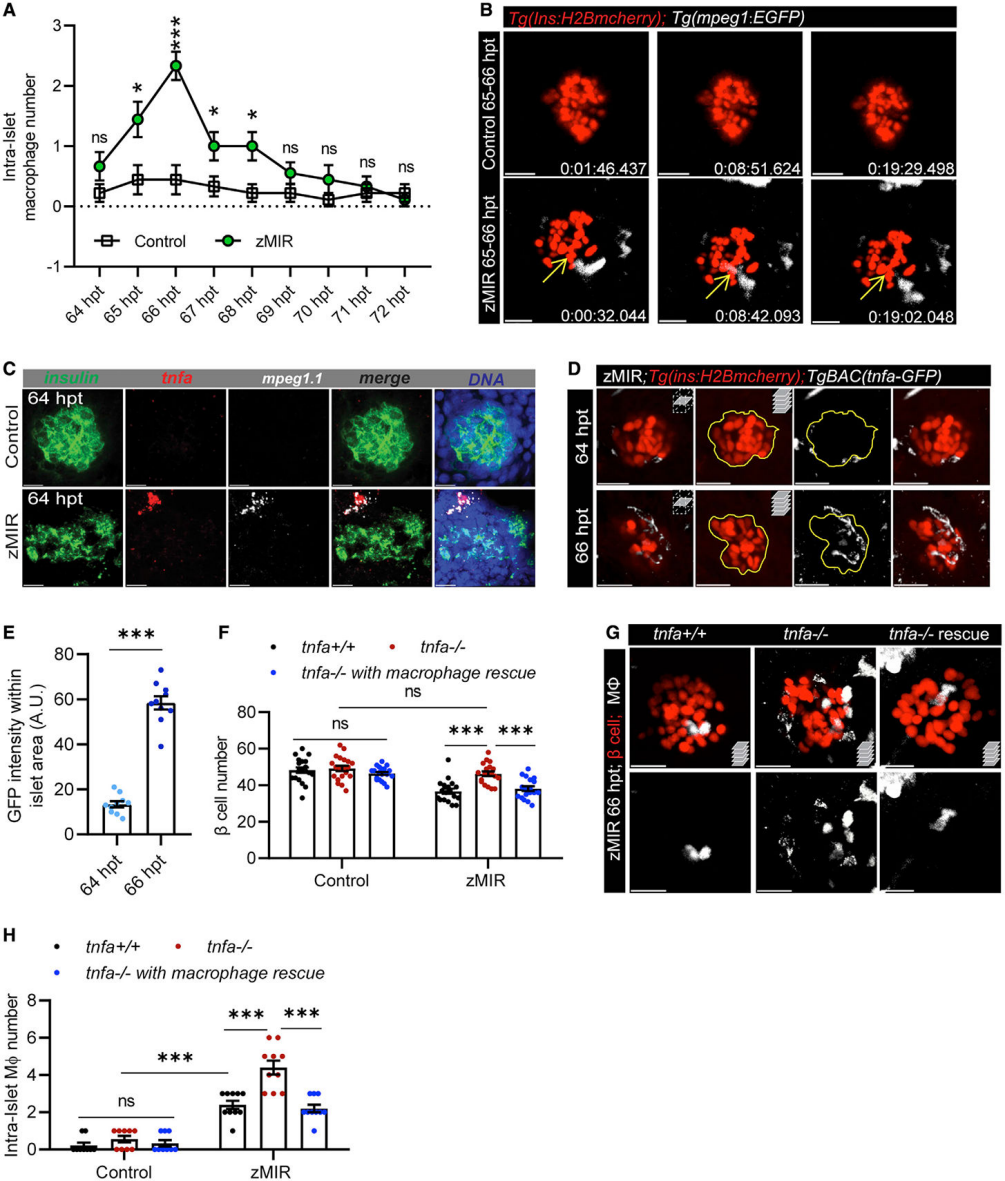
Macrophage-derived Tnfa is necessary for β cell loss in zMIR fish (A) Intra-islet macrophage number in zMIR fish from 64–72 h based on 30 s/frame time-lapse videos hourly. Data represent means ± SEM, n ≥ 10 per group, multiple t tests; *p < 0.05, ***p < 0.001. (B) Macrophage surveillance of the principal islet during 65–66 hpt in zMIR and control fish, showing that macrophage-contacted β cells remained intact. Live imaging videos were recorded at 30-s intervals. Yellow arrows indicate a macrophage-contacted β cell. Scale bars, 20 μm. (C) Representative RNAscope images of *insulin*, *mpeg1.1*, *tnfa* expression at 64 h in control and zMIR fish. Scale bars, 20 μm. (D) Islet images of *TgBAC(tnfa:GFP)*, *Tg(ins:H2B-mCherry)*, zMIR fish at 64 and 66 h. The GFP signal was detected by immunofluorescence. Scale bars, 20 μm. (E) Quantification of GFP signals in the islet area (outline in D). Data are mean ± SEM, n = 10/group, unpaired t test; bar graphs represent mean ± SEM; ***p < 0.001. (F) The effect of *tnfa* re-expression in macrophages on β cell loss in zMIR fish. Data are mean ± SEM, n > 15/group, two-way ANOVA followed by Tukey’s multiple comparisons test; ***p < 0.001. (G) Representative images showing increased intra-islet macrophages in zMIR fish with different tnfa genotypes (^+/+^, ^−/−^, and ^−/−^ with macrophage rescue). Red marks β cells, and white labels macrophages. Scale bars, 10 μm. (H) Quantification of intra-islet macrophages in zMIR fish with different *tnfa* genotypes (^+/+^, ^−/−^, and ^−/−^ with macrophage rescue) at 66 hpt. Data are mean ± SEM, n = 10 per group, two-way ANOVA followed by Tukey’s multiple comparisons test; ***p < 0.001.

Macrophages can kill live cells by phagocytosis (Brown and Neher, 2012; Feng et al., 2018). To test whether macrophages directly kill β cells, we used live imaging to visualize macrophage-β cell interactions. Macrophages started to interact with the principal islet in zMIR animals at 65 hpt. The surveillance peaked at 67 hpt and returned to baseline by 69 hpt (Figure 2A). In contrast, very few macrophages were found to interact with the islet in the controls (Figure 2A). The time course of macrophage surveillance coincided with the time course of β cell loss (Figure 1B). In some cases, intra-islet macrophages contacted or even wrapped around β cells (Yang et al., 2020). These findings are consistent with a direct role of macrophages in β cell loss.

However, the total numbers of intra-islet macrophages were low (2 per islet) during 65–67 hpt (Figure 2A), and they resided within the islet for only a short duration (about 20 min) (Figure 2B). The β cells remained intact after macrophage departure (Figure 2B), and β cell decline continued after the peak of macrophage surveillance (Figure 1B). These results argue against β cell loss by macrophage phagocytosis. Thus, we investigated whether macrophage-derived factors induce β cell loss.

Macrophages can cause β cell death by releasing proinflammatory cytokines, such as TNFA (Donath et al., 2008). Therefore, we assessed the expression of *tnfa* in macrophages of zMIR fish. Using RNAscope, we determined that peri- and intra-islet macrophages (identified by an *mpeg1* probe) were the only cells that express *tnfa* (as indicated by a *tnfa* probe) at 64 hpt in zMIR larvae (Figure 2C), suggesting that these macrophages are proinflammatory (Nguyen-Chi et al., 2015). Using a transgenic reporter of *tnfa* expression, *TgBAC(tnfa:EGFP)* (Marjoram et al., 2015), we detected a marked increase in cells expressing *tnfa* in the islet vicinity from 64–66 hpt (Figures 2D and 2E). There was an inverse correlation between the macrophage-β cell distance and the expression levels of *tnfa* in macrophages (Figures S2B and S2C). These results suggest that stressed β cells recruit macrophages and induce *tnfa* expression.

To determine the role of Tnfa, we inactivated *tnfa* by generating frameshift mutations using CRIPSR-Cas9 (Figure S2D). We chose to characterize mutants carrying a 59-bp deletion encompassing the 3′ portion of the first coding exon and 5′ portion of the downstream intron. RT-PCR analysis confirmed the predicted mutant transcript and detected a minor product because of activation of a cryptic splice donor 10 bp upstream of the deletion (Figure S2D). The minor cDNA is thus 64 bp smaller than the wild type (WT). The major mutant transcript is predicted to only generate the N-terminal 41 residues of Tnfa, followed by 24 gibberish amino acids. The product lacks all annotated trimerization sites and receptor binding sites. A minor transcript is predicted to generate a nonfunctional product with 38 residues of Tnfa at the N terminus, followed by 10 random residues. This is therefore a null mutation. Loss of *tnfa* function had little effect in non-zMIR siblings but prevented overnutrition-induced β cell loss (Figure 2F). The *tnfa* mutant zMIR fish also had normal whole-body free glucose content after overnutrition (Figure S2E). There were significantly more intra-islet macrophages in the *tnfa* mutant zMIR fish (Figures 2G, 2H, and S2F), indicating that Tnfa plays a direct or indirect role in limiting the number of macrophages in the islet. These results demonstrate that *tnfa* is required for β cell loss in zMIR fish after overnutrition.

We hypothesized that Tnfa is required specifically in macrophages to mediate β cell loss. To test this, we generated transgenic lines that specifically express Tnfa in macrophages (driven by an *mpeg1* promoter) (Figure S2G) and found that, although the transgene had no detectable effect on β cell number or glucose homeostasis in sibling controls, it restored β cell loss and glucose dyshomeostasis in *tnfa*^*−/−*^ zMIR animals (Figures 2F and S2E). Rescue of *tnfa* expression in macrophages also reduced the number of intra-islet macrophages to control levels (Figures 2G, 2H, and S2F). These results demonstrate that macrophage-derived Tnfa is sufficient for inducing β cell loss.

### Tnfa induces *cxcl8a* expression in zebrafish and mammalian β cells under ER stress

**Figure 3.**
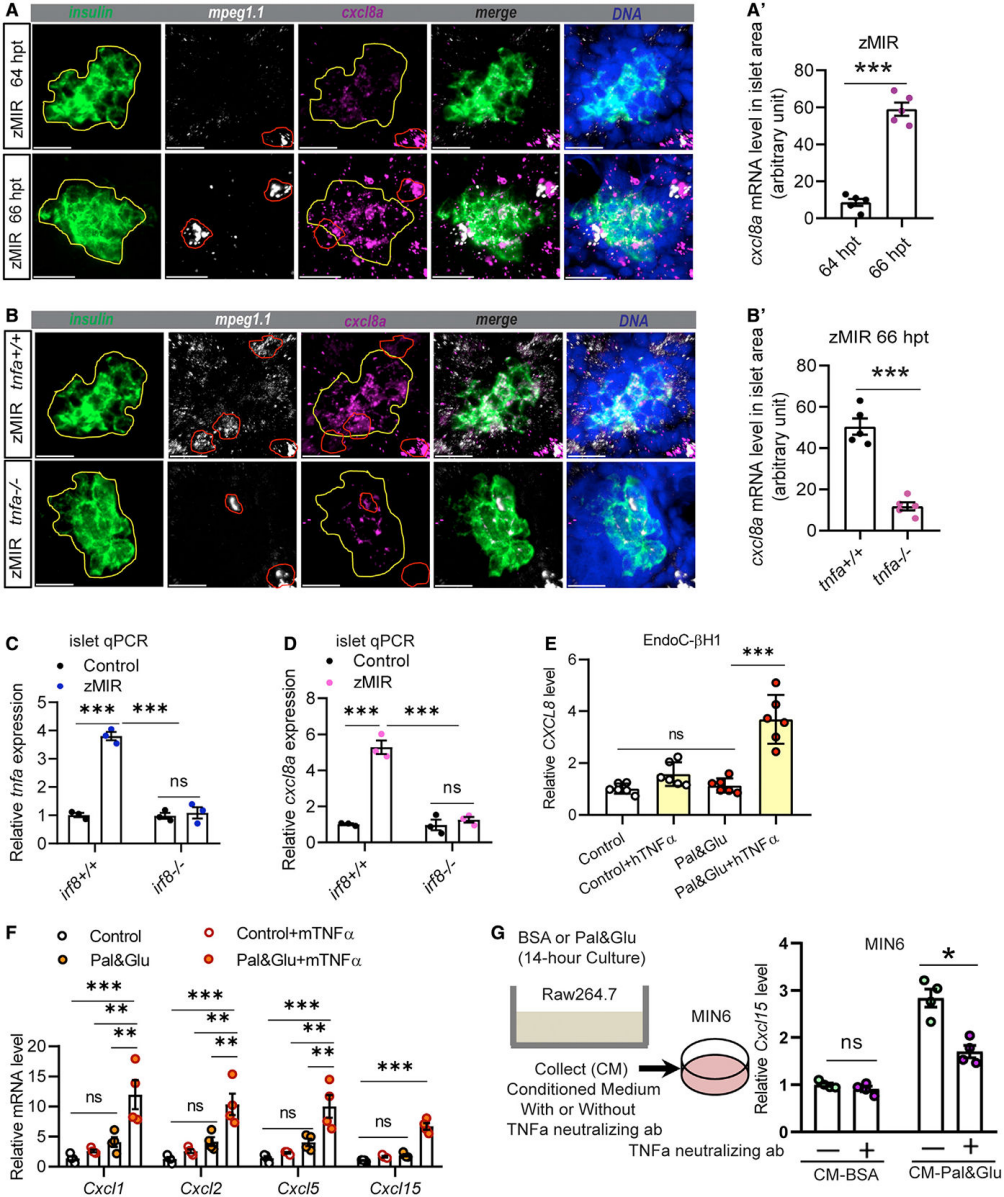
A conserved TNFA-*CXCL8* axis in β cells under ER stress (A) Representative RNAscope images of *insulin*, *mpeg*, and *cxcl8a* expression at 64 and 66 h zMIR fish. A yellow line in each image outlines the islet, and a red line outlines macrophages. Scale bars, 20 μm. (A′) Quantification of *cxcl8a* signals in the islet area (outlined in yellow) from RNAscope images at 64 and 66 h in zMIR fish. Unpaired t test; bar graphs represent mean ± SEM; n = 5/group, ***p < 0.001. (B) Representative RNAscope images of *insulin*, *mpeg*, and *cxcl8a* expression at 66 h in *tnfa*^−/−^, zMIR and control zMIR fish. Scale bars, 20 μm. (B′) Quantification of *cxcl8a* signals in the islet area (outlined in yellow) from RNAscope images at 66 h in *tnfa*^−/−^, zMIR, and control zMIR fish. Unpaired t test; bar graphs represent mean ± SEM; n = 5/group; ***p < 0.001. (C) Islet qRT-PCR analysis of *tnfa* at 66 hpt in *irf8*^−/−^, zMIR, and control zMIR fish. Data are mean ± SEM; n = 3/group; two-way ANOVA followed by Tukey’s multiple comparisons test; ***p < 0.001. (D) Islet qRT-PCR analysis of islet *cxcl8a* expression at 66 hpt in *irf8*^−/−^, zMIR, and control zMIR fish. Data are mean ± SEM. n = 3/group, two-way ANOVA followed by Tukey’s multiple comparisons test; ***p < 0.001. (E) Effect of TNFA treatment on *CXCL8* expression in EndoC-H1 cells cultured under high-palmitate (0.5 mM) and glucose (25 mM) conditions (pal&glu). Data are mean ± SEM. n = 6/group, one-way ANOVA followed by Tukey’s multiple comparisons test; ***p < 0.001. (F) Effect of TNFA treatment on expression of *Cxcl1*, *Cxcl2*, *Cxcl5*, and *Cxcl15* in MIN6 cells under pal&glu conditions. Data are mean ± SEM. n = 4/group, one-way ANOVA followed by Tukey’s multiple comparisons test; **p < 0.01, ***p < 0.001. (G) Effect of the TNFA-neutralizing antibody on *Cxcl15* expression in MIN6 cells cultured in Raw264.7 conditioned medium. The schematic shows generation of Raw264.6 conditioned medium for MIN6 cells. Neutralized TNFA significantly decreases induction of *cxcl15* expression under conditioned medium in MIN6 cells. Data are mean ± SEM. n = 4/group, multiple t tests; *p < 0.05.

Tnfa may change gene expression in β cells to induce cell loss. We have reported previously that *cxcl8a* was increased in the zMIR islet at 64 hpt (Yang et al., 2020). CXCL8, or interleukin-8 (IL-8), is a chemokine that activates CXCR1 and CXCR2 to induce chemotaxis (Matsushima et al., 2022). To determine the cell type(s) in which this induction occurs, we performed multiplex RNAscope analysis. The results indicated that, at 64 hpt, *cxcl8a* was highly expressed in peri-islet macrophages (identified by expression of *mpeg1*) but poorly expressed in β cells (identified by *insa* expression), consistent with previous results (Yang et al., 2020; Figure 3A and 3A′). At 66 hpt, when β cell loss accelerated, *cxcl8a* expression increased significantly in β cells (Figures 3A and 3A′). Quantitative reverse-transcriptase polymerase chain reaction (qRT-PCR) analysis of islet RNAs also showed increased *cxcl8a* expression at 66 hpt compared with 64 hpt (Figure S3A).

We next determined the role of macrophages and Tnfa in *cxcl8a* induction. Compared with control zMIR fish, induction of *cxcl8a* was diminished in β cells of *tnfa*^*−/−*^ zMIR fish (Figures 3B and 3B′), which was further verified by qRT-PCR (Figure S3B). As expected, islets from macrophage-deficient *irf8*^*−/−*^ zMIR fish had significantly decreased expression of *tnfa* at 66 hpt (Figure 3C). They also had significantly diminished expression of *cxcl8a* (Figure 3D). Thus, macrophage-derived Tnfa is required for induction of *cxcl8a* expression in islet β cells in zMIR fish.

Although *Cxcl8* expression has been shown to be induced by TNFA in other cell types in mammals (Bezzerri et al., 2011; Namba et al., 2017), whether it occurs in β cells is unknown. To investigate the conservation of *Cxcl8* induction by TNFA in mammalian β cells, we turned to cell culture models. Proliferating EndoC-βH1 cells have been increasingly used as human β cell models *in vitro* (Scharfmann et al., 2014). These cells had low *CXCL8* expression in normal and glucolipotoxic (0.5 mM palmitate and 25 mM glucose) medium. When recombinant human TNFA was added to glucolipotoxic medium, these cells had significantly increased *CXCL8* expression (Figure 3E). Similar results were seen in an immortalized mouse β cell line, MIN6. Because *Cxcl8* is absent from the mouse genome, we investigated its functional analogs, which activate CXCR1 and CXCR2, including *Cxcl1*, *Cxcl2*, *Cxcl5*, and *Cxcl15* (Shibata et al., 2013). Glucolipotoxic medium caused a small increase in transcripts of these chemokines, which was markedly enhanced by recombinant TNFA (Figure 3F). These data suggest that TNFA is sufficient to induce expression of CXCR1/2 ligands in mammalian β cells cultured in high-glucose and high-lipid media that cause strong ER stress (Yang et al., 2020).

We then used macrophage and β cell lines to further study their interaction *in vitro*. When exposed to glucolipotoxicity, Raw264.7 macrophage cells and MIN6 β cells had a small increase in *Tnfa* and *Cxcl15* expression (Figures S3C-S3F). When they were co-cultured at a 100:1 ratio (MIN6:Raw264.7), glucolipotoxicity caused a much stronger induction of *Tnfa* and *Cxcl15* expression (Figures S3E and S3F). Knockdown of *Tnfa* mRNA in Raw264.7 cells by Cas13d-mediated degradation using different sgRNAs (Wessels et al., 2020) (Figure S3G) reduced *Cxcl15* expression in the co-culture according to the efficiency of the sgRNAs (Figure S3H). Raw264.7 conditioned medium also induced higher *Cxcl15* expression in MIN6 cells, which was blunted by a TNFA-neutralizing antibody (Figure 3G). These results demonstrate that macrophage-derived TNFA is necessary and sufficient to induce chemokine expression in mammalian β cells under conditions of ER stress.

### ER stress is required for *cxcl8a* induction and in zMIR fish

**Figure 4.**
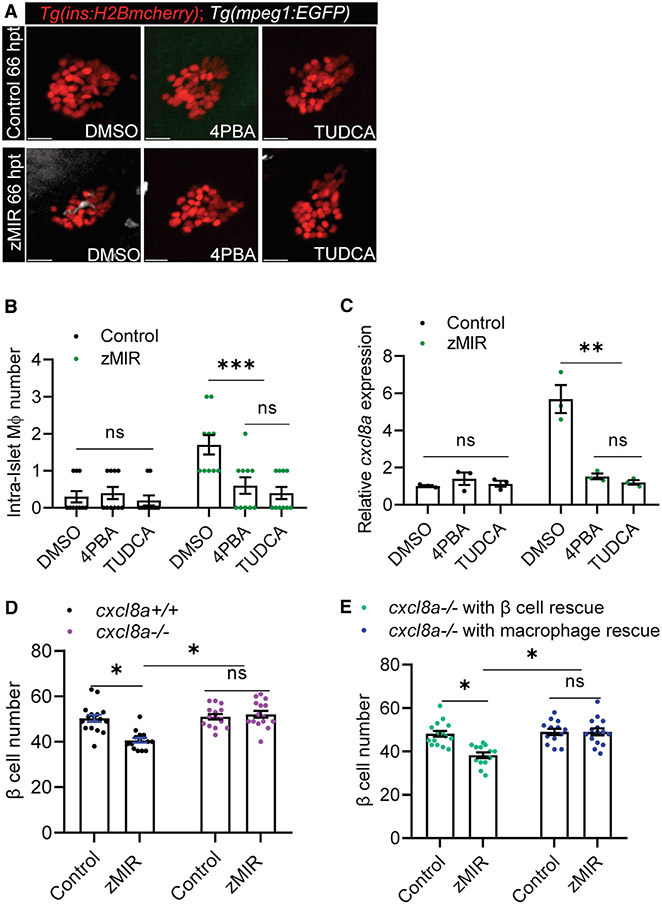
β cell-derived Cxcl8a is required for β cell loss in zMIR fish (A) Representative images of intra-islet macrophages in control and zMIR fish treated with DMSO (vehicle), TUDCA, and 4-PBA at 66 hpt. Red cells are islet β cells, and white cells are macrophages. Scale bars, 10 μm. (B) Intra-islet macrophage numbers in control and zMIR fish treated with DMSO (vehicle), TUDCA, and 4-PBA at 66 hpt. Data are mean ± SEM. n = 10/group, two-way ANOVA followed by Tukey’s multiple comparisons test; ***p < 0.001. (C) Islet qRT-PCR analysis of *cxcl8a* at 66 hpt in control and zMIR fish treated with DMSO (vehicle), TUDCA, and 4-PBA at 66 hpt. Data are mean ± SEM. n = 3/group, two-way ANOVA followed by Tukey’s multiple comparisons test; **p < 0.01. (D) β Cell numbers in zMIR fish with or without *cxcl8a* function. Data are mean ± SEM. n = 15/group, two-way ANOVA followed by Tukey’s multiple comparisons test; *p < 0.05. (E) β Cell numbers in *cxcl8a*^−/−^, zMIR fish with *cxcl8a* rescue in β cells or macrophages. The rescue was achieved by *Tg(ins:cxcl8a-P2A-nEGFP)* and *Tg(mpeg1:cxcl8a-P2A-tagRFPcaax)*, respectively. Data are mean ± SEM. n > 10/group, two-way ANOVA followed by Tukey’s multiple comparisons test; *p < 0.05.

In our previous study, we have shown that ER stress mediates β cell loss through activation of RIPK3, which initiates a proinflammatory gene expression program in islet β cells, leading to macrophage recruitment to the islet. ER stress is necessary for β cell loss in zMIR (Yang et al., 2020). We confirmed that treating zMIR fish with the chemical chaperones 4-phenylbutyric acid (4-PBA) and tauroursodeoxycholic acid (TUDCA) during the second and third sessions of overnutrition prevented β cell loss (Figures S4A and S4B). These treatments also decreased intra-islet macrophage numbers at 66 hpt (Figures 4A and 4B). These treatments also significantly decreased *tnfa* transcripts in the zMIR islet (Figure S4C). Treatment with 4-PBA or TUDCA also abolished *cxcl8a* induction (Figure 4C). These results indicate that ER stress is required for *cxcl8a* induction in islet β cells.

To determine whether Cxcl8a is required for β cell loss in the zMIR model, we generated frameshift mutations in the *cxcl8a* locus using CRIPSR-Cas9 (Figure S4D; Jao et al., 2013). We characterized mutants harboring a mutation consisting of a 28-bp deletion and a 12-bp insertion. RT-PCR analysis confirmed the mutant transcript and detected no aberrant splicing (Figure S4D). The product of the mutant transcript is predicted to consist of the N-terminal 68 residues of Cxcl8a, followed by 33 gibberish amino acids. The mutant Cxcl8a protein thus lacks the C-terminal 29 residues important for dimerization and oligomerization as well as receptor binding. Peptides lacking the C-terminal 27 amino acids of human CXCL8 have a 68-fold increase in K_d_ to human neutrophils and 56-fold increase in half maximal effective concentration (EC50) in releasing elastase from human neutrophils compared with a fully active peptide (Clore et al., 1990). The mutation is therefore predicted to be near-complete loss of function. This mutation significantly decreases coronary endothelial cell proliferation during cardiac regeneration after injury (El-Sammak et al., 2022). Loss of *cxcl8a* function in zMIR fish prevented β cell loss (Figure 4D). To dissect whether the function of Cxcl8a from macrophages differs from that from β cells, we generated transgenic lines that specifically express *cxcl8a* in the macrophage (directed by an *mpeg1* promoter) or β cell lineage (directed by an *insa* promoter) (Figures S4E and S4F). Re-expression of *cxcl8a* in β cells, but not macrophages, restored overnutrition-induced β cell loss in *cxcl8*^*−/−*^ zMIR fish (Figure 4E). These results suggest that β cell-derived Cxcl8a is essential for β cell loss.

### Cxcl8a from β cells is necessary for recruiting neutrophils to the islet

**Figure 5.**
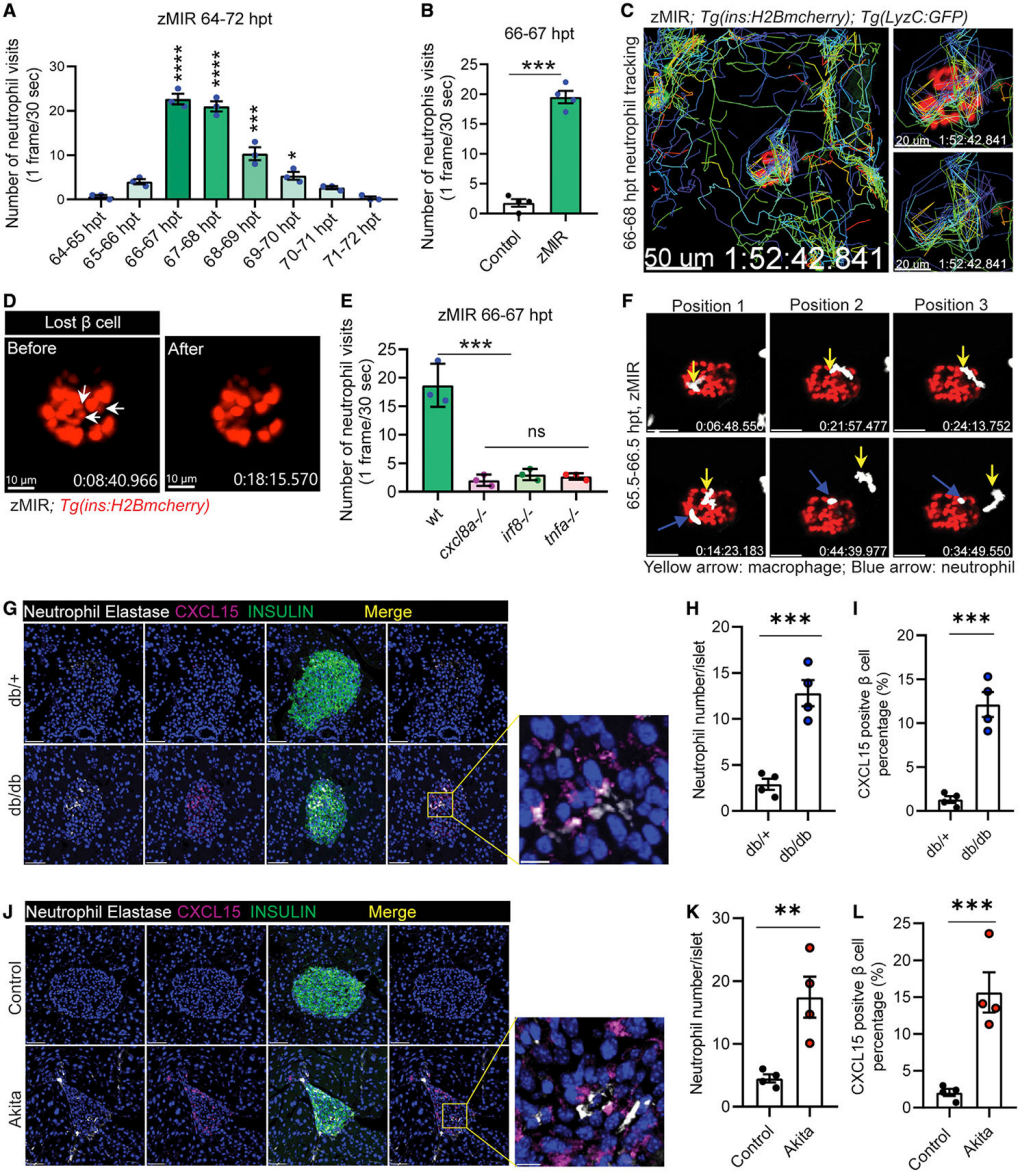
Cxcl8a is necessary for recruiting neutrophils to the islet (A) Quantification of islet visits by neutrophils in live imaging videos from 64–72 h. Videos were recorded at 30 s/frame. Data are mean ± SEM. n = 3/group, one-way ANOVA followed by Tukey’s multiple comparisons test; *p < 0.05, ***p < 0.001, ****p < 0.0001. (B) Quantification of islet visits by neutrophils in 30 s/frame live imaging videos of control and zMIR fish from 66–67 h. Unpaired t test; n = 4/group; bar graphs represent mean ± SEM; ***p < 0.001. See also Videos S1A and S1B. (C) Representative tracks of neutrophil movement in the islet vicinity in a zMIR fish from 66–68 h. Scale bars, 50 μm. See also Video S2. (D) β cell loss during the 67–68 hpt interval in a representative islet. White arrows point to the β cells lost during the interval. Scale bars, 15 μm. See also Video S3C. (E) Quantification of islet visits by neutrophils in live imaging videos of *tnfa*^−/−^, zMIR fish; *irf8*^−/−^, zMIR fish; *cxcl8a*^−/−^, zMIR fish, and control zMIR fish from 66–67 h. Videos were recorded at 30 s/frame. Data are mean ± SEM. n = 3/group, one-way ANOVA followed by Tukey’s multiple comparisons test; ***p < 0.001. (F) Representative video frames showing neutrophils tracking a macrophage in the principal islet in zMIR fish from 66–67 hpt. Yellow arrows point to macrophages, and blue arrows point to neutrophils. Videos were recorded at 30-s intervals. Macrophages were distinguished from neutrophils by their morphological irregularity and lower velocity. See also Video S3D. (G) Representative immunofluorescence images of neutrophil elastase and CXCl15 in pancreas sections of 8-week-old *db*/+ (B6.BKS(D)-Leprdb/J) and *db/db* mice. Scale bars, 50 μm. Inset: contact between neutrophils and CXCl15-positive β cells. Scale bars, 10 μm. (H) Quantification of intra-islet neutrophil numbers of *db*/+ and *db/db* mice (n = 4; at least 50 islets were quantified in each animal). Unpaired t test, bar graphs represent mean ± SEM, ***p < 0.001. (I) Quantification of CXCL15-positive β cells in *db*/+ and *db/db* mice (n = 4; at least 50 islets were quantified in each animal). Unpaired t test, bar graphs represent mean ± SEM, ***p < 0.001. (J) Representative immunofluorescence images of neutrophil elastase and CXCl15 in pancreas sections of 8-week-old control (C57BL/6) and Akita (C57BL/6-Ins2Akita/J) mice. Scale bars, 50 μm. Inset: contacts between neutrophils and CXCl15-positive β cells. Scale bars, 10 μm. (K) Quantification of intra-islet neutrophil numbers of control and Akita mice (n = 4; at least 50 islets were quantified in each animal). Unpaired t test, bar graphs represent mean ± SEM, **p < 0.01. (L) Quantification of CXCL15-positive β cells in control and Akita mice (n = 4; at least 50 islets were quantified in each animal). Unpaired t test, bar graphs represent mean ± SEM, ***p < 0.001.

We next explored the mechanism by which Cxcl8a mediates β cell loss. CXCL8 is a potent activator and chemoattractant for neutrophils (Baggiolini et al., 1989; De Oliveira et al., 2013). To determine whether neutrophils are recruited to the islet in response to *cxcl8a* induction, we generated transgenic lines that express EGFP or tagRFP specifically in neutrophils. Live imaging showed that neutrophils surveyed the principal islet between 66 and 70 hpt in zMIR fish, starting about 1 h after the beginning of macrophage recruitment (Figure 5A). Neutrophils “visited” the islet in zMIR fish much more frequently within 66–67 hpt than control fish (Figure 5B; Videos S1A and S1B). To better inspect the neutrophil-β cell interaction and its consequences, we imaged at shorter time intervals (15 s) for a longer duration (2 h) (Figure 5C; Video S2). Neutrophils repeatedly visited a few hotspots in the principal islet (Figure 5C). β Cell loss was observed near these hotspots, as indicated by the disappearance of the H2B-mCherry signal (Video S3A). To determine whether these hotspots were exit points for neutrophils, we performed live imaging in fish with GFP-labeled endothelium (*Tg(flk:GFP)*). We were unable to determine the exit points of neutrophils because of limitations of the microscope (Video S3B). The relationship between neutrophil visits and β cell loss was apparent even when both cell types were labeled with red fluorescence (Figure 5D; Video S3C). We did not observe nuclear fragments in the β cells, indicating non-apoptotic cell loss. These results implicate neutrophils in β cell loss.

We next determined whether *cxcl8a* is necessary for neutrophil recruitment. The number of neutrophil visits was significantly decreased in *cxcl8a*^*−/−*^ zMIR fish compared with control zMIR fish (Figure 5E). Consistent with *cxcl8a* being induced by macrophage-derived Tnfa, the number of neutrophil visits was also decreased significantly in zMIR fish that were deficient in *irf8* or *tnfa* (Figure 5E). Similarly, pharmacological relief of ER stress also significantly decreased the number of neutrophil visits (Figure S5A). These results indicate that Cxcl8a is necessary for neutrophil chemotaxis to the principal islet.

Live imaging results also provided a clue for selection of hotspots. In instances where macrophages and neutrophils were present during live imaging, we found that the fast-moving neutrophils usually visited areas where a slow-moving ameba-like macrophage had surveilled previously (Figures 5F and S5B; Videos S3D and S3E). These results suggest that macrophage contact may mark β cells for neutrophil surveillance.

The recruitment of macrophages and neutrophils in the zMIR islet during β cell loss prompted us to examine these innate immune cells in 2 non-autoimmune mouse diabetes models, C57BLKS/J(BKS) db/db (db/db for short) and Akita. Both models display misfolded proinsulin and a small but significant increase in β cell death starting in young adults (Arunagiri et al., 2019; Hodish et al., 2010; Puff et al., 2011; Shirakawa et al., 2013). As reported previously (Ehses et al., 2007), the number of islet macrophages was increased in db/db mice compared with db/+ littermates. In 8-week-old db/db and Akita mice (Figures S5C and S5D), the number of islet macrophages was increased compared with their control littermates (Figures S5E, S5E′, S5F, and S5F′). The number of islet neutrophils, identified by expression of neutrophil elastase, was also increased in db/db (Figures 5G and 5H) and Akita (Figures 5J and 5K) mice. Accompanying the neutrophils was an increase in CXCL15-positive β cells in db/db (Figure 5I) and Akita islets (Figure 5L), similar to the increase in Cxcl8a chemokine in the zMIR islet. These results suggest a role of islet macrophages and neutrophils in β cell loss in db/db and Akita mice.

### Islet neutrophils are necessary for β cell loss

**Figure 6.**
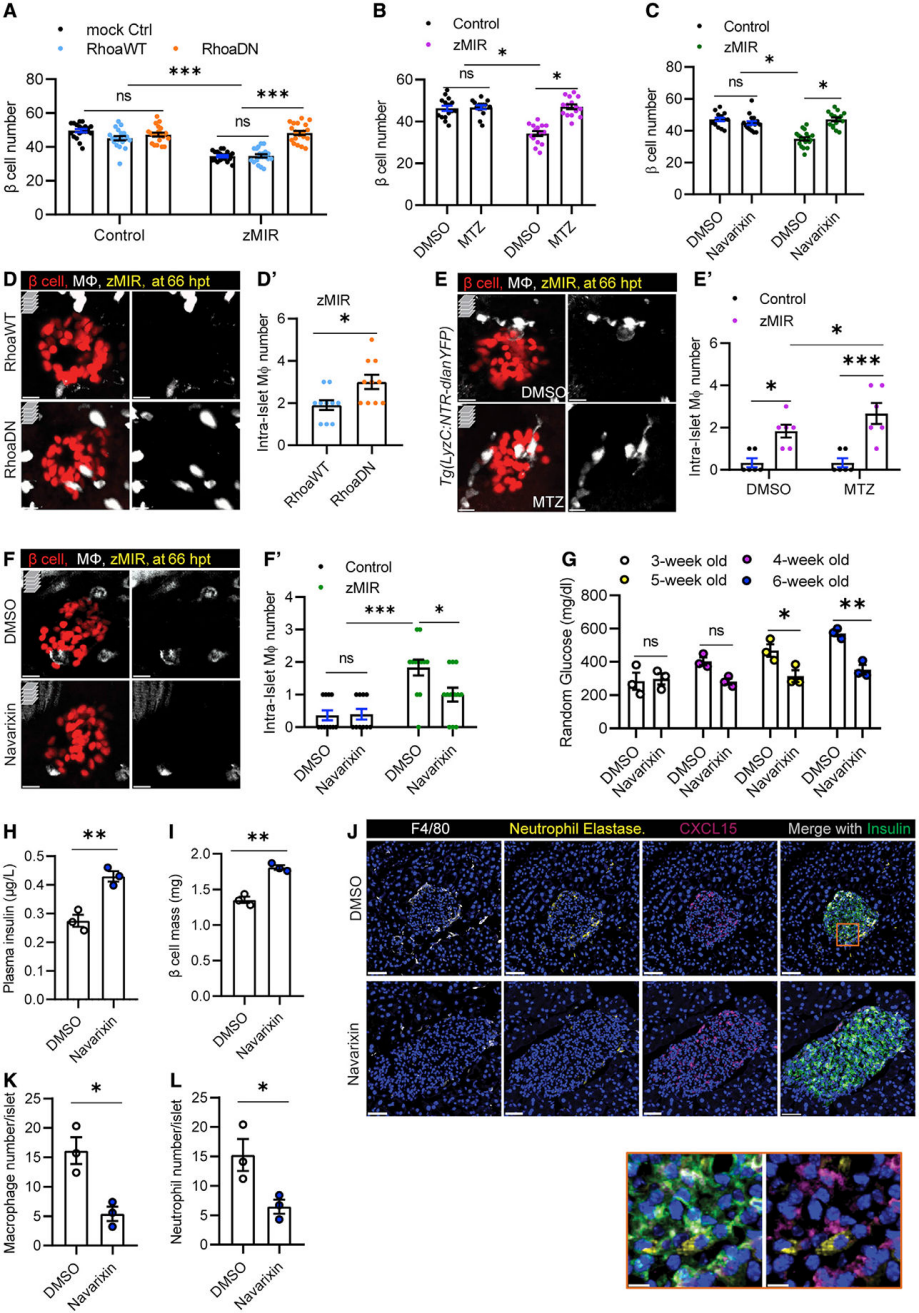
Islet neutrophils are necessary for β cell loss (A) Effect of impairing neutrophil motility on β cell loss. Data are mean ± SEM. n > 10/group, two-way ANOVA followed by Tukey’s multiple comparisons test; ***p < 0.001. (B) Effect of MTZ-NTR-mediated neutrophil ablation on β cell loss. Data are mean ± SEM. n > 10/group, two-way ANOVA followed by Tukey’s multiple comparisons test; *p < 0.05. (C) Effect of the Cxcr1/2 antagonist on β cell loss. Data are mean ± SEM. n > 10 per group, two-way ANOVA followed by Tukey’s multiple comparisons test; *p < 0.05. (D) Representative images of intra-islet macrophages in zMIR fish with *Tg(LyzC:mCherry-2a-RhoaDN)* (labeled RhoaDN) or *Tg(LyzC:mCherry-2a-Rhoa^WT^)* (labeled RhoaWT) at 66 hpt, showing β cells (red) and macrophages (white). Scale bars, 10 μm. (D′) Quantification of intra-islet macrophages in zMIR fish with *Tg(LyzC:mCherry-2a-RhoaDN)* or *Tg(LyzC:mCherry-2a-Rhoa^WT^)*. Unpaired t test, n = 10, bar graphs represent mean ± SEM, *p < 0.05. (E) Representative images of intra-islet macrophages in zMIR, *Tg(LyzC:NTR-dlanYFP)* fish treated with DMSO or MTZ (2.5 mM) at 66 hpt, showing β cells (red) and macrophages (white). Scale bars, 10 μm. (E′) Quantification of intra-islet macrophages in zMIR fish with or without neutrophil depletion. Data are mean ± SEM. n = 6/group, two-way ANOVA followed by Tukey’s multiple comparisons test; *p < 0.05, ***p < 0.001. (F) Representative images of intra-islet macrophages in control fish and zMIR fish at 66 hpt after treatment with DMSO or navarixin (10 μM), showing β cells (red) and macrophages (white). Scale bars, 10 μm. (F′) Quantification of intra-islet macrophages in zMIR fish treated with vehicle or navarixin. Data are mean ± SEM. n = 10/group, two-way ANOVA followed by Tukey’s multiple comparisons test; *p < 0.05, ***p < 0.001. (G) Weekly random blood glucose levels in Akita (*C57BL/6-Ins2Akita/J*) mice with daily peritoneal injection of DMSO or navarixin (5 μg/g), starting at 3 weeks of age (day 21). Unpaired t test, n = 3/group, bar graphs represent mean ± SEM; *p < 0.05, **p < 0.01. (H) Plasma insulin levels in Akita (*C57BL/6-Ins2Akita/J*) mice after 3 weeks of daily injections of DMSO or navarixin (5 μg/g). Unpaired t test, n = 3/group, bar graphs represent mean ± SEM, **p < 0.01. (I) β Cell mass of Akita (*C57BL/6-Ins2Akita/J*) mice after 3 weeks of injection with DMSO or navarixin (5 μg/g). Unpaired t test, n = 3/group, bar graphs represent mean ± SEM, **p < 0.01. (J) Representative immunofluorescence images of neutrophil elastase, F4/80, insulin, and CXCl15 in pancreas sections of Akita (C57BL/6-Ins2Akita/J) mice after 3 weeks of daily injections of DMSO or navarixin (5 μg/g). Scale bars, 50 μm. Inset: contacts between neutrophils and CXCL15-positive β cells. Scale bars, 10 μm. (K) Quantification of intra-islet neutrophil numbers of Akita (*C57BL/6-Ins2Akita/J*) mice after 3 weeks of daily injections of DMSO or navarixin (n = 3; at least 50 islets were quantified in each animal). Unpaired t test, bar graphs represent mean ± SEM, *p < 0.05. (L) Quantification of intra-islet macrophage numbers in pancreas sections of Akita (*C57BL/6-Ins2Akita/J*) mice after 3 weeks of daily injections of DMSO or navarixin (n = 3; at least 50 islets were quantified in each animal). Unpaired t test, bar graphs represent mean ± SEM, *p < 0.05.

We then determined whether neutrophils are required for β cell loss in zMIR fish using three independent approaches to impair or ablate neutrophils. First we generated a transgenic line that biscistronically expressed WT (RhoA^WT^) or dominant-negative RhoA (RhoA^DN^) and mCherry in neutrophils. As expected, RhoA^DN^ impaired neutrophil motility and their chemotaxis to wounds compared with neutrophils expressing RhoA^WT^ and mCherry or mCherry alone (Figures S6A-S6C). Overnutrition did not cause β cell loss in zMIR fish with impaired neutrophil motility (Figure 6A). Impairing neutrophil chemotaxis also increased the number of intra-islet macrophages in zMIR fish compared with zMIR fish with Rhoa^WT^ expression in neutrophils (Figures 6D and 6D′). Second, we generated zMIR fish that expressed NTR specifically in the neutrophil lineage for selective ablation (Figures S6D and S6E). Ablation of neutrophils prevented β cell loss (Figure 6B). It also increased the number of intra-islet macrophages (Figures 6E and 6E′). Finally, we used a CXCR1/2 antagonist, navarixin, to block Cxcl8a signaling and neutrophil migration (Coombs et al., 2019; Oehlers et al., 2015; Figures S6F and S6F′). When applied to zMIR fish during 56–72 hpt, navarixin prevented β cell loss (Figure 6C). In contrast to the neutrophil-specific manipulations above, navarixin decreased the number of intra-islet-macrophages (Figures 6F and 6F′), probably by inhibiting macrophage chemotaxis. These results indicate that functional neutrophils are necessary for overnutrition-induced β cell loss in zMIR fish.

Using navarixin, we assessed whether innate immune cell recruitment to the islet plays a role in diabetes progression in male Akita mice. Daily injection of navarixin starting at 3 weeks of age did not affect weight gain or cause overt adverse effects (Figures S6G and S6H). Navarixin treatment significantly slowed the rise of random blood glucose compared with Akita mice injected with vehicle (Figure 6G). After 3 weeks, navarixin-treated Akita mice had significantly higher random blood insulin levels (Figure 6H) and increased β cell mass (Figure 6I) compared with vehicle-treated controls. As expected, navarixin treatment decreased the number of macrophages (Figures 6J and 6K) and neutrophils (Figures 6J and 6L) compared with vehicle treatment, similar to zMIR fish. However, it did not significantly change CXCL15-positive β cells (Figure S6I). These results indicate that islet macrophages and neutrophils play a role in development of diabetes in Akita mice.

## DISCUSSION

The pathogenesis of T2D involves β cell loss and persistent ER stress (Eizirik et al., 2020; Sahin et al., 2021; Shrestha et al., 2021). Although persistent ER stress induces β cell death *in vitro* and *ex vivo*, how it causes β cell loss *in vivo* is not clear. We have shown previously that overnutrition in a zMIR model causes β cell-specific ER stress. ER stress in these cells triggers Ripk3-mediated induction of il1b, leading to macrophage recruitment and subsequent β cell loss and glucose dyshomeostasis (Yang et al., 2020). In this work, we address the cellular mechanisms that drive β cell loss and identify a complex intercellular communication involving macrophages, neutrophils, and the stressed β cells themselves. We also demonstrate that macrophages and neutrophils are increased in 2 non-autoimmune mouse models of diabetes and play a role in pathogenesis.

We showed that macrophages are necessary for overnutrition-induced β cell loss in zMIR fish. Macrophages were recruited to the islet of zMIR fish at the time of β cell loss. Chemical relief of ER stress prevented macrophage recruitment, demonstrating a critical role of ER stress. Depletion of macrophages using 3 different approaches prevented β cell loss (Figures 1D-1F and 1H). Macrophage recruitment by β cells has been reported in other zebrafish models, including β cell-specific overexpression of il1b (Delgadillo-Silva et al., 2019) and il1b-tnfa-ifng (Ibrahim et al., 2020) and β cell-specific oxidative stress (Kulkarni et al., 2018). Macrophages have also been shown to be necessary for β cell loss in ZDF rats (Jourdan et al., 2013). In ZDF rats, depleting islet macrophages also decreases expression of several inflammation-related genes, including *Tnfa* (Jourdan et al., 2013). We demonstrated that *tnfa* is increasingly expressed in macrophages as they move closer to the principal islet in zMIR fish (Figure 2D), suggesting that it is induced by islet-derived factors. Inactivating *tnfa* abolished β cell loss, which was negated by transgenic rescue in macrophages (Figure 2F), indicating that Tnfa is a critical mediator of macrophage-mediated β cell loss.

We identified the Cxcl8 family of chemokines as important downstream mediators of Tnfa. In zMIR fish, induction of *cxcl8a* in β cells occurs after recruitment of macrophages and depends on macrophages and *tnfa* (Figure 3B). In db/db and Akita mice, CXCL15, a functional equivalent of CXCL8, is increased in β cells at the early stages of diabetes (Figures 5I-5L), concomitant with an increase in islet macrophages. TNFA likely acts directly on β cells to induce *CXCL8* family chemokines because recombinant TNFA induces their expression in cultured human and mouse β cells (Figures 3E and 3F). The results also suggest that increased expression of *CXCL8*-family chemokines is a conserved β cell response to TNFA.

Remaining important questions include what signal(s) attract(s) neutrophils to islet hotspots and how they are generated. It is conceivable that neutrophils target severely stressed or dysfunctional β cells. RNAscope results indicate that *cxcl8a* levels are uneven among β cells (Figures 3A and 3B). Because CXCL8 promotes not only chemotaxis but also phagocytic function of neutrophils (Beste et al., 2015), the relative levels of Cxcl8a may be a component of the signal. Neutrophils seem to be attracted to macrophage-contacted sites in the islet (Figures 5F and S5B), suggesting that the physical interaction generates a signal for neutrophils. This signal may be Cxcl8a or other, currently unidentified molecules.

We demonstrated that neutrophils are necessary for overnutrition-induced β cell loss. In zMIR fish, neutrophils visit hotspots in the islet, where macrophages make contact and β cell loss occurs (Figures 5F and S5B). Neutrophil recruitment requires β cell-derived Cxcl8a. Impairing neutrophil motility or depleting neutrophils prevented β cell loss (Figures 6A-6C). We showed that ER stress is necessary for cxcl8a induction and neutrophil recruitment in the zMIR model (Figures 4C and S5A). In 2 mouse models of diabetes with non-autoimmune β cell death involving ER stress, db/db and Akita mice, islet neutrophils are increased at an early stage of diabetes development, which, to our knowledge, has not been reported before. Although neutrophils have not been implicated previously in T2D, they are known to play a role in pathogenesis of T1D (Huang et al., 2016). In non-obese diabetic (NOD) mice, for instance, islet neutrophil recruitment is critical for initiation of diabetes (Diana et al., 2013). Blocking neutrophil recruitment by CXCR1/2 antagonists dampens or reverses diabetes development (Citro et al., 2015; Diana and Lehuen, 2014). Similarly, treating zMIR fish and Akita mice with a CXCR1/2 antagonist also suppresses diabetes development (Figures 6C-6I). However, neutrophils cause β cell loss indirectly in NOD mice by perpetuating insulitis (Diana et al., 2013). In contrast, neutrophils seem to be directly involved in β cell loss in zMIR fish, as suggested by the close spatial and temporal relationship, although the exact mechanism requires further investigation. Increased expression of *Cxcl8* family members has been reported in β cells from T2D mouse models and human donors (Cnop et al., 2005; Jörns et al., 2006; Nunemaker et al., 2014). It is possible that a similar increase in islet neutrophils may be found in these samples. Our study indicates that neutrophils play a critical role in the pathogenesis of non-autoimmune diabetes.

Our study demonstrates that overnutrition-induced β cell death requires intricate β cell-macrophage-neutrophil communication. Targeting these signaling pathways may preserve β cell function and prevent β cell loss.

### Limitations of the study

There are some limitations of our study. The key observations are made in larvae of a zebrafish model of muscle insulin resistance with chronic overnutrition. This was necessary for live imaging of the islet in its native environment. The observed islet inflammation in zMIR fish seems to be transitory, and β cell death occurs during a small window of time. Such acute changes may be difficult to recapitulate in other models. Extending the relevance of these findings to adult humans, although necessary, may be even more challenging. In confirmation of the zebrafish results, we provided evidence that recombinant TNFA is sufficient to induce CXCL8 or its equivalents in mammalian β cells *in vitro*, suggesting conservation of these signaling cascades. We found increased macrophages and neutrophils in the islets of mouse models of diabetes, consistent with a role of β cell-macrophage-neutrophil crosstalk in diabetes pathogenesis in mammals.

## STAR★METHODS

### RESOURCE AVAILABILITY

#### Lead contact

Further information and requests for resources and reagents should be directed to and will be fulfilled by the Lead Contact, Wenbiao Chen (wenbiao.chen@vanderbilt.edu).

#### Materials availability

All zebrafish lines generated in this study are available upon request.

#### Data and code availability

Data reported in this paper will be shared by the lead contact upon request.

This paper does not report original code.

Any additional information required to reanalyze the data reported in this paper is available from the lead contact upon request.

### EXPERIMENTAL MODEL AND SUBJECT DETAILS

#### Cell culture

The murine MIN6 immortalized β cell line was cultured in standard conditions (Miyazaki et al., 1990). Briefly, MIN6 cells were obtained from Addexbio and grown in AddexBio Advanced DMEM Medium (C0003-04) containing 15% FBS, 0.05 mM 2-mercaptoethanol, and penicillin (100 U/mL)/streptomycin (100 mg/mL). Cells were cultured at 37°C with 5%CO2. Murine RAW264.7 macrophages were obtained from the ATCC (T1B-71) and cultured at recommended conditions. Immortalized human β cell lines EndoC-H1 and EndoC-H2 cells were kindly provided from Dr. Roland Stein (Vanderbilt University). They were propagated in DMEM (Gibco and Thermo Fisher Scientific, Waltham, MA) in presence of 5.6 mmol/L glucose, 2% BSA (Serologicals Proteins, Kankakee, IL), 100 μU/mL penicillin, 100 μg/mL streptomycin, 50 μmol/L 2-mercaptoethanol, 10 mmol/L nicotinamide, 5 μg/mL transferrin, and 6.7 ng/mL sodium selenite (Sigma-Aldrich, St. Louis, MO).

#### Zebrafish

Zebrafish were raised in an Aquatic-Habitats system on a 14-/10-h light/dark cycle. Embryos were raised at 28.5°C in an incubator on a 14-/10-h light/dark cycle. Embryos were obtained by natural cross and kept in embryo rearing solution and staged according to standard methods. Transgenic lines used in this study were *Tg(ins:H2Bmcherry)* (Maddison and Chen, 2012), *Tg(actc1b:dnig-f1ra-EGFP)* or zMIR (Maddison et al., 2015), *Tg(mpeg1:EGFP)* (Felix et al., 2011), *TgBAC(tnfa:GFP)* (Marjoram et al., 2015), *Tg(mpeg1:GAL4)*; *Tg(UAS-E1b:NTR-mCherry)* (Davison et al., 2007; Felix et al., 2011), *Tg(LyzC:GFP)* (Hall et al., 2007), *Tg(LyzC:dsRed2)* (Hall et al., 2007), *Tg(mfap4:tdTomato-CAXX)*^*xt6*^ (Oehlers et al., 2015), *Tg(LyzC:NTR-dlanYFP)* (Oehlers et al., 2015). The zebrafish used for this study were around 9 dpf, before sex can be identified. All animal studies were approved by the Vanderbilt Institutional Animal Care and Use Committee.

#### Mouse

All strains of mice in these studies were maintained in the facility under a 12-h light cycle with free access to standard chow pellets and water unless specified. All mice used for mating were between 2–6 months old. Each mouse used was genotyped by tail DNA PCR amplification at the age between 18–20 days. Male Ins2^+/Akita^ mice were peritoneally injected daily with either Navarixin (diluted with DMSO at 5μmg/mL) in a 5 mg/kg of body weight or DMSO (equal volume) started from day 21. Mice were weighed every day and random glucose levels were monitored every week. Blood was collected for plasma Insulin levels and pancreas were sampled and weighed for β cell mass and Immunofluorescence. For full list of genotyping primers, see Supplemental Table. All animal studies were approved by the Vanderbilt Institutional Animal Care and Use Committee.

### METHOD DETAILS

#### Establishment and identification of transgenic lines and mutation lines

The Tol2 transposon system (Suster et al., 2009) was used to generate the *Tg(LyzC:mCherry-2a)*, *Tg(LyzC:mCherry-2a-Rhoa*^*WT*^*), Tg(LyzC:mCherry-2a-Rhoa*^*DN*^*), Tg(mpeg1:P2A-tagRFP*_*caax*_*), Tg(ins:cxcl8a-P2A-nEGFP), Tg(mpeg1:tnfa-P2A-tagRFP_caax_)* transgenic lines in this study. Briefly, 1 nL of solution containing 20 pg of transgene plasmid and 50 pg of Tol2 mRNA was injected into each zebrafish zygote. Mutations in *tnfa*, *cxcl8a* were generated using CRISPR-Cas9 as described previously using recombinant Cas9 (PNA Bio) (Yin et al., 2015). Typically, each zygote is injected with 1 nL solution containing 2 μM Cas9 and 2.5 μM sgRNA. Two knockout lines in each gene were characterized for an initial cross-validation before selecting one line for all the experiments. For genotyping of the mutant fish, briefly, a small part of tail fin was cut from individual fish for DNA extraction and PCR amplification (Genotyping primers, Star table). Then PCR products were resolved by gel electrophoresis in a 3% agarose gel. *Irf8*^*(ST95)*^ (Shiau et al., 2015) was a gift from Will Talbot (Stanford).

#### Overnutrition and compound treatment

Overnutrition was achieved by culturing larvae in 5% chicken egg yolk solution for 8 h as described (Yang et al., 2020). For multiple days of overnutrition, larvae were rinsed and kept in nutrient-free 0.3× Danieau buffer for 16 h before the next session of overnutrition treatment. Compounds used were metronidazole (MTZ, 2.5mM) and Navarixin (CXCR1/2 antagonist, 10μM).

#### Liposome delivery

For macrophage depletion studies, larvae were anesthetized in MESAB and then injected transpericardially with 5 nL clodronate liposomes or control liposomes (Encapsula Nano Sciences) 24 h prior to experimentation.

#### Immunostaining and quantification of β cells and intra-islet macrophage number in zebrafish

Immunostaining, preparation of slides and quantification of the number of β-cell nuclei were done as previously published using transgenic line expressing a nuclear localized mCherry (Maddison and Chen, 2012). For quantification of intra-islet macrophage number, zebrafish were first euthanized with ice-cold egg water and then fixed with 4% PFA at 66 hpt. Briefly, fixed samples were dehydrated in 100% methanol, rehydrated with PBST and permeabilized with 100% acetone at −20°C for 30 min. After washing with PBST, the permeabilized tissues were blocked in 5% FBS/PBST at room temperature for 1 h followed by overnight incubation with primary antibody at 4°C. After washing with PBST 3 times, secondary antibody was added followed by incubation at room temperature for 2 h. Confocal microscopy was done using either an LSM780 or LSM880 confocal microscope using a 20x, 40x, or 63× objective. Images were processed in the Zen Blue (Zeiss) and Imaris.

#### Total glucose assay

Total glucose was determined using the Amplex Red Glucose/Glucose Oxidase Assay Kit. A pool of 10 larvae was homogenized in 100 μL of sample buffer. The homogenate was spun at 13,000 rpm for 5 min. Free glucose in 10 μL of supernatant (equivalent of one larva) was determined according to the manufacturer’s instructions. Fluorescence (excitation, 535 nm; emission, 590 nm) was measured using a SpectraMax M5 Microplate Reader. At least five pools of each experimental group were measured.

#### Zebrafish islet isolation

For islet isolation, larvae with *Tg(ins:H2B-mCherry)* were euthanized in ice-cold water and suspended in a solution of HBSS with 50 μg/mL Liberase DH, lightly crushed with a pestle and incubated at 37°C for 2 min. RPMI with 10% FCS was added to stop the reaction and the entire solution placed in a 10 cm Petri dish containing RPMI with 10% FCS. Islets were picked manually under a fluorescent stereomicroscope and placed into a 6cm dish with RPMI, and this process was repeated to limit extraneous tissue.

#### Live imaging

Eight-day-old zebrafish larvae that had been through overnutrition protocol were found to be highly susceptible to prolonged immobilization. Prolonged immobilization for more than 2 h would result in either abolished phenotype (macrophage infiltration and β cell loss) or death. For all live imaging studies, larvae were immobilized right side up in 1.5% low melting agarose in a glass-bottomed dish. The dish was covered with 0.3% Danieau’s solution containing 0.01 mg/mL Tricaine (Ethyl 3-aminobenzoate methanesulfonate). Live imaging was performed on an inverted laser scanning confocal system, ZEISS LSM 780. The climate chamber covering the microscope stage was set at temperatures 28°C. In the *Tg(ins:H2B-mCherry), Tg(mpeg1:EGFP), Tg(LyzC:RFP)* triple-transgenic animals, the GFP, mCherry and RFP signals were acquired simultaneously using the 488 nm and 561 nm laser lines, respectively. In the *Tg(ins:H2B-mCherry); Tg(LyzC:GFP)* double transgenic animals, the GFP and mCherry signals were acquired simultaneously using the 488 nm and 561 nm laser lines. Volumetric time-lapse videos were recorded at 15s or 30s intervals with a Z-step thickness of 1.2μm and an average total thickness of 35μm, and an XY resolution of 0.12 μm per pixel (512 × 512 pixels). Laser power was maintained as low as possible (<1.5%) to minimize phototoxicity.

#### Establishment of *tnfa* knockdown raw264.7

For Cas13d mediated *Tnfa* knockdown in raw264.7 cells, the lentiviral Cas13d expressing vector was purchased from Addgene (Addgene#138147). Two sgRNAs were designed through a web-based application (https://cas13design.nygenome.org/). To construct individual sgRNA vectors, annealed oligonucleotide pairs were ligated to BsmBI-cleaved Cas13d expressing backbones (hU6-DR_BsmBI-EFS-RfxCas13d-NLS-2A-Puro-WPRE). After verification by Sanger sequencing, the Cas13d-sgRNA^Tnfa^ constructs, along with pMD2.G and psPAX2, were transfected into low passage HEK293T cells with CalPhos Mammalian Transfection Kit. After overnight incubation, the cells were washed with PBS and replenished with pre-warmed DMEM medium. Conditioned medium was harvested 48 h later, and cellular debris was filtered out using 0.45 μm PVDF filter. To assess the knockdown efficiency of each *Tnfa* sgRNA, raw 264.7 cells were transduced with the lentiviral medium. Transduced cells were selected with 5 μg/mL puromycin starting at 24 h post-transduction. After 4 days of selection, cells were harvested for total RNA extraction and gene expression analysis.

#### Co-culture and conditioned medium experiment

For co-culture experiment, MIN6 (5 × 10^5^) and raw264.7 (Mock-ctr, *Tnfa*-kd1, *Tnfa*-kd2) (5 × 10^3^) cells (MIN6: raw264.7 = 100:1) were seeded in 6-well plate containing DMEM supplemented with 10% FBS and either 500 μM palmitate and 25mM glucose or vehicle (BSA) for 14 h. To assess the effects of medium conditioned by raw264.7 cells, (Mock-ctr, *Tnfa*-kd1, *Tnfa*-kd2), 1 × 10^6^ cells were incubated in 6 mm dishes containing DMEM supplemented with 10% FBS and either 500 μM palmitate and 25mM glucose or vehicle (BSA). After 14 h incubation, conditioned medium from raw264.7 were collected and neutralizing antibodies TNFA were then only added to the conditioned medium (10ng/mL) (from control). MIN6 cells were incubated in the resultant mediums for 14 h. To test whether TNFA could induct cxcl15 in MIN6 or cxcl8 in EndoC-H2 under high palmitate and glucose condition, 10ng/mL recombinant mouse TNFA Protein or recombinant human TNFA Protein were added to MIN6 or EndoC-H2 with the presence of 500 μM palmitate and 25mM glucose.

#### RNA extraction and qPCR

For zebrafish islet qPCR, at least 70 islets were pooled directly into a tube with TRIZOL (Thermo) for RNA extraction. RNA was isolated using Direct-Zol columns and concentrated using RNA Clean and Concentrator −25 columns. RNA was reverse transcribed using oligo-dT and MMLV reverse transcriptase. The cDNAs were subjected to PCR on a BioRad CFX96 machine and amplicons were detected using SYBR green. For cell culture, cells were washed two times with cold PBS prior to lysis by TRIZOL reagent and the total RNA reverse transcribed using oligo-dT and MMLV reverse transcriptase. Levels of expression were determined by the Pfaffl method (Pfaffl, 2001). All the qPCR primers used in this study are listed in Table S1.

#### RNAscope and *in situ* hybridization

Zebrafish larvae were fixed at hour 64 or 66 and embedded into O.C.T. for cryosectioning. Islet-containing frozen sections (12 μm) were selected for *in situ* hybridization using the RNAscope 2.0 High-Definition kit according to the manufacturer’s instructions. Probes hybridization and signal amplification were performed according to the manufacturer’s instructions. The signal for each probe was revealed by Opal 520, Opal 570, and Opal 690 according to the ACD manual. After the final wash with wash buffer reagents from ACD, slides were mounted with ProLong Diamond, and images were acquired on a Zeiss LSM880. Images were analyzed by Imaris. For quantification of *cxcl8a* transcripts within islet area, five z-stacks spanning the entire depth of the islet area (40× objective) were analyzed (n = 5/per group). Images were processed in ImageJ software using the Threshold function, followed by quantification using Fiji’s Analyze Particles program on maximum intensity projections. The *cxcl8a* transcripts (magenta puncta) were counted manually using Fiji’s Cell Counter program while moving through z-stacks.

#### Immunofluorescence of antibodies and β cell mass in mice

For sectioning, tissues were dissected in ice-cold 1X PBS, immediately followed by PBS washes, and fixed in 4% paraformaldehyde (PFA)/PBS, overnight at 4°C. Then, the tissues were dehydrated in gradient sucrose/PBS solution, then embedded in Tissue-Tek* OCT in −80°C until sectioning. Ten-micrometer-thick frozen sections were cut and stained. Primary antibodies used were listed in the Table. Images were obtained using LSM880 confocal microscope.

For β-cell mass assessment, ~2% of each pancreas was immunolabeled and analyzed (5–10 sections/animal, each separated by 250 μm). Slides were scanned at 20× magnification under Scan Bright Fluorescent Scope System. β cell mass was quantified from scanned tissue sections immunolabeled for insulin using HALO software. HALO discriminates between stained/unstained area and thus can quantitate the area of the pancreas occupied by insulin.

### QUANTIFICATION AND STATISTICAL ANALYSIS

For multiple group comparisons, one-way or two-way analysis of variance (ANOVA) test was performed, and a post hoc Tukey’s multiple comparisons test was used. A p value less than 0.05 was considered statistically significant. Values represent means ± SEM. Analyses were done using GraphPad Prism.

## Supplementary Material

1

2

3

4

## Figures and Tables

**Table:** KEY RESOURCES TABLE

REAGENT or RESOURCE	SOURCE	IDENTIFIER
Antibodies		
Guinea pig anti-Insulin	Dako	Cat# A0564; RRID:AB_10013624
InVivoMAb anti-mouse F4/80	Bio X Cell	Cat#74262001; RRID: AB_10949019
Anti-GFP antibody	Abcam	Cat# ab13970; RRID:AB_300798
Neutrophil Elastase	SANTA CRUZ BIOTECHNOLOGY	Cat# sc-55549; RRID:AB_831596
TNF alpha Monoclonal Antibody (TN3-19.12)	Invitrogen	Cat# 14-7423-81; RRID:AB_468491
GFP Monoclonal Antibody (3E6)	Invitrogen	Cat# A11120; RRID:AB_221568
CXCL15/Lungkine Polyclonal Antibody	Bioss	Cat# bs-2554R; RRID:AB_10856560
Cy2-AffiniPure Donkey Anti-Chicken IgY(IgG)	CiteAb	Cat# 703-225-155; RRID: AB_2340370
Alexa Fluor™ 488 goat anti-rabbit IgG(H + L)	Invitrogen	Cat# A11008; RRID:AB_143165
Alexa Fluor™ 647 goat anti-guinea pig IgG(H + L)	Invitrogen	Cat# A21450; RRID:AB_141882
Alexa Fluor™ 568 goat anti-rat IgG(H + L)	Invitrogen	Cat# A11077; RRID:AB_2534121
Alexa Fluor™ 488 goat anti-guinea pig IgG(H + L)	Invitrogen	Cat# A11073; RRID:AB_2534117
Alexa Fluor™ 647 goat anti-rabbit IgG(H + L)	Invitrogen	Cat# A21244; RRID:AB_2535812
Chemicals, peptides, and recombinant proteins		
Palmitic acid	Sigma-Aldrich	Cat# P5585
Metronidazole	Sigma-Aldrich	Cat# M1547
Low melting agarose	Sigma-Aldrich	Cat# A9414
Ethyl 3-aminobenzoate methanesulfonate	Sigma-Aldrich	Cat# E10521
Navarixin (CXCR1/2 antagonist)	APExBIO	Cat# A3802
Trizol	Invitrogen	Cat# 15596-026
DMEM	Thermo Fisher Scientific	Cat# 11995065
SYBR™ Green PCR Master Mix	Thermo Fisher Scientific	Cat# 4309155
Mouse TNF-alpha Recombinant Protein	eBioscience	Cat# 29-8321-65
Human TNF-alpha Recombinant Protein	Invitrogen	Cat# A42552
Clodronate	ClodronateLiposomes.com	Cat# F70101-AH
Hoechst 33342 Staining Dye Solution	Invitrogen	Cat# H3570
Critical commercial assays		
RNAscope® Fluorescent Multiplex Detection Reagents	Advanced Cell Diagnostics	Cat# 320851
RNAscope® Probe- Dr-cxcl8a-C2	Advanced Cell Diagnostics	Cat# 522981-C2 NCBI:XM_001342570.7
RNAscope® Probe- Dr-ins-C1	Advanced Cell Diagnostics	Cat# 437781-C1 NCBI:NM_131056.1
RNAscope® Probe- Dr-tnfa-C2	Advanced Cell Diagnostics	Cat# 575111-C2 NCBI:NM_212859.2
RNAscope® Probe- Dr-mpeg1.1-C3	Advanced Cell Diagnostics	Cat# 536171-C3 NCBI:NM_212737.1
Opal 520	Akoya Biosciences	Cat#FP1487001KT
Opal 570	Akoya Biosciences	Cat#FP1488001KT
Opal 690	Akoya Biosciences	Cat#FP1497001KT
Standard Macrophage Depletion Kit	Clodrosome®	Cat#CLD-8901
Amplex™ Red Glucose/Glucose Oxidase Assay Kit	Invitrogen	Cat# A22189
Direct-zol RNA Miniprep Kits	Zymo Research	Cat# R2050
RNA Clean & Concentrator-5	Zymo Research	Cat# R1013
GoScript™ Reverse Transcription System	Promega	Cat# A5000
PVDF filter	Millipore	Cat# SLHVM33RS
Liberase DH	Sigma-Aldrich	Cat# 5401119001
CalPhos™ Mammalian Transfection Kit	Takara	Cat# 631312
Cas9 Protein	PNA Bio	Cat# CP01
Experimental models: Cell lines		
Mouse MIN-6 cell	Roland Stein lab	N/A
Human EndoC-H1 cell	Roland Stein lab	N/A
Mouse RAW264.7 cell	ATCC	Cat# T1B-71
Experimental models: Organisms/strains		
Zebrafish: *Tg(actc1b:dnigf1ra-EGFP)*, zMIR	Maddison et al., (2015)	N/A
Zebrafish: *Tg(ins:H2BmCherry)*	Maddison and Chen (2012)	N/A
Zebrafish: zMIR, *Tg(ins:H2BmCherry)*	Maddison et al. (2015)	N/A
Zebrafish: *Tg(mpeg1:EGFP)*	Ellett et al., (2011)	N/A
Zebrafish: zMIR, *Tg(mpeg1:EGFP)*	This paper	N/A
Zebrafish: *Tg(mpeg1:GAL4)*; *Tg(UAS-E1b:NTR-mcherry)*	Ellett et al., (2011); Davison et al. (2007)	N/A
Zebrafish: *TgBAC(tnfα:GFP)*	Marjoram et al., (2015)	N/A
Zebrafish: *Tg(LyzC:GFP)*	Hall et al. (2007)	N/A
Zebrafish: *Tg(LyzC:dsRed2)*	Hall et al. (2007)	N/A
Zebrafish: *Tg(mfap4:tdTomato-CAXX)^xt6^*	Oehlers et al., (2015)	N/A
Zebrafish: *Tg(LyzC:NTR-dlanYFP)*	Oehlers et al., (2015)	N/A
Zebrafish: zMIR, *Tg(mpeg1:GAL4); Tg(UAS-E1b:NTR-mCherry)*	This paper	N/A
Zebrafish: zMIR, *Tg(ins:H2BmCherry); Tg(mpeg1:EGFP), Tg(LyzC:dsRed2)*	This paper	N/A
Zebrafish: zMIR, *Tg(ins:H2BmCherry); Tg(mpeg1:EGFP), Tg(LyzC:GFP)*	This paper	N/A
Zebrafish: zMIR, *irf8*−/−, *Tg(ins:H2BmCherry); Tg(mpeg1:EGFP), Tg(LyzC:dsRed2)*	This paper	N/A
Zebrafish: zMIR, *tnfa*−/−, *Tg(ins:H2BmCherry) Tg(mpeg1:EGFP), Tg(LyzC:dsRed2)*	This paper	N/A
Zebrafish: zMIR, *cxcl8a*−/−, *Tg(ins:H2BmCherry) Tg(mpeg1:EGFP), Tg(LyzC:dsRed2)*	This paper	N/A
Zebrafish: *Tg(LyzC:mCherry-2a)*	This paper	N/A
Zebrafish: *Tg(LyzC:mCherry-2a-Rhoa^WT^)*	This paper	N/A
Zebrafish: *Tg(LyzC:mCherry-2a-Rhoa^DN^)*	This paper	N/A
Zebrafish: *Tg(mpeg1: cxcl8a-P2A-tagRFP_caax_)*	This paper	N/A
Zebrafish: *Tg(ins:cxcl8a-P2A-nEGFP)*	This paper	N/A
Zebrafish: *Tg(mpeg1:tnfα-P2A-tagRFP_caax_)*	This paper	N/A
Mouse: C57BL/6-Ins2^Akita^/J	The Jackson Laboratory	JAX 003548
Mouse: B6.BKS(D)-Lepr^db^/J	The Jackson Laboratory	JAX 000697
Oligonucleotides		
Cas9-sgRNA *tnfa* sgrna1: ggTATGGAGCGTGAAGCAGA	This paper	N/A
Cas9-sgRNA *tnfa* sgrna2: ggTAGACTGGAGAGATGACC	This paper	N/A
Cas9-sgRNA *cxcl8a* sgrna1: ggTTCAATGCAGCGACAGCG	This paper	N/A
Cas9-sgRNA *cxcl8a* sgrna2: ggGTCCAGTTGTCATCAAGG	This paper	N/A
Cas13d-sgRNA (mouse) *tnfa* sgRNA1: AGTAGACAAGGTACAACCCATCG	This paper	N/A
Cas13d-sgRNA (mouse) *tnfa* sgRNA2: AAGTTCAGTAGACAGAAGAGCGT	This paper	N/A
*cxcl8a* genotyping primer Forward: GCTTTCAGGAATGAGCTTGAGAG	This paper	N/A
*cxcl8a* genotyping primer Reverse: TCTTAACCCATGGAGCAGAGG	This paper	N/A
*tnfa* genotyping primer Forward: ATCTTCAAAGTCGGGTGTATGG	This paper	N/A
*tnfa* genotyping primer Reverse: CTCACCACTTCCATCTTGTTGA	This paper	N/A
*irf8* genotyping primer Forward: GCTGCTGTTTGTAACGGCATAC	This paper	N/A
*irf8* genotyping primer Reverse: CGCTTACTTTGAAAATGGACGC	This paper	N/A
*tnfa* RT-PCR primers Forward: TATGAAGCTTGAGAGTCGGGC	This paper	N/A
*tnfa* RT-PCR primers Reverse: GTGCAGCTGATGTGCAAAGA	This paper	N/A
*cxcl8a* RT-PCR primers Forward: CTGAACTGAGCTCCTCAGGAAT	This paper	N/A
*cxcl8a* RT-PCR primers Reverse: GTGATCCGGGCATTCATGGT	This paper	N/A
Ins2^Akita^ genotyping primers Forward: TGCTGATGCCCTGGCCTGCT	JAX protocol	olMR1093
Ins2^Akita^ genotyping primers Reverse: TGGTCCCACATATGCACATG	JAX protocol	olMR1094
Lepr^db^ genotyping primers Forward: AGAACGGACACTCTTTGAAGTCTC	JAX protocol	oIMR0985
Lepr^db^ genotyping primers Reverse: CATTCAAACCATAGTTTAGGTTTGTGT	JAX protocol	oIMR0986
Recombinant DNA		
hU6-DR_BsmBI-EFS-RfxCas13d-NLS-2A-Puro-WPRE	Addgene	Cat# 138147
Software and algorithms		
GraphPad Prism 8	GraphPad Software	https://www.graphpad.com/
Imaris v 7.5x	Bitplane	http://www.bitplane.com/
SpectraMax M5 Microplate Reader	Molecular Devices	https://www.moleculardevices.com/products/microplate-readers
ImageJ	https://doi.org/10.1038/nmeth.2089	https://imagej.nih.gov/ij/
HALO® IMAGE ANALYSIS PLATFORM	Indica labs	https://indicalab.com/halo
Zeiss LSM Imaging Software (confocal)	Carl Zeiss	http://www.zeiss.com; RRID:SCR_014344
Aperio ScanScope slide scanner	Aperio, Vista, CA	https://www.leicabiosystems.com/digital-pathology/scan/aperio

## Highlights

- Macrophages are recruited by stressed β cells but do not directly cause β cell loss
- Macrophage-derived TNFA induces CXCL8 expression in β cells
- CXCL8 attracts neutrophils to islet “hotspots,” leading to β cell loss
- Neutrophil numbers are increased in murine models of non-autoimmune diabetes
