# Supplementary material for: Macrophages and neutrophils are necessary for ER stress-induced β cell loss
Source: Cell Rep. Author manuscript; Available in PMC 2022 Sep 5. (PMC9444341; doi:10.1016/j.celrep.2022.111255)
Supplement: 1 [file NIHMS1832200-supplement-1.pdf]

Cell Reports, Volume *40*

Supplemental information

Macrophages and neutrophils are necessary

for ER stress-induced β cell loss

Bingyuan Yang, Liu Yang, Yueyang Wang, Lisette A. Maddison, Zihan Tang, Sander Haigh, Yulong Gong, Yue Zhang, Brittney A. Covington, Karin J. Bosma, Xin Tong, Patrick Page-McCaw, Maureen Gannon, Qing Deng, and Wenbiao Chen

SUPPLEMENTARY INFORMATION

Supplemental Figure Legend

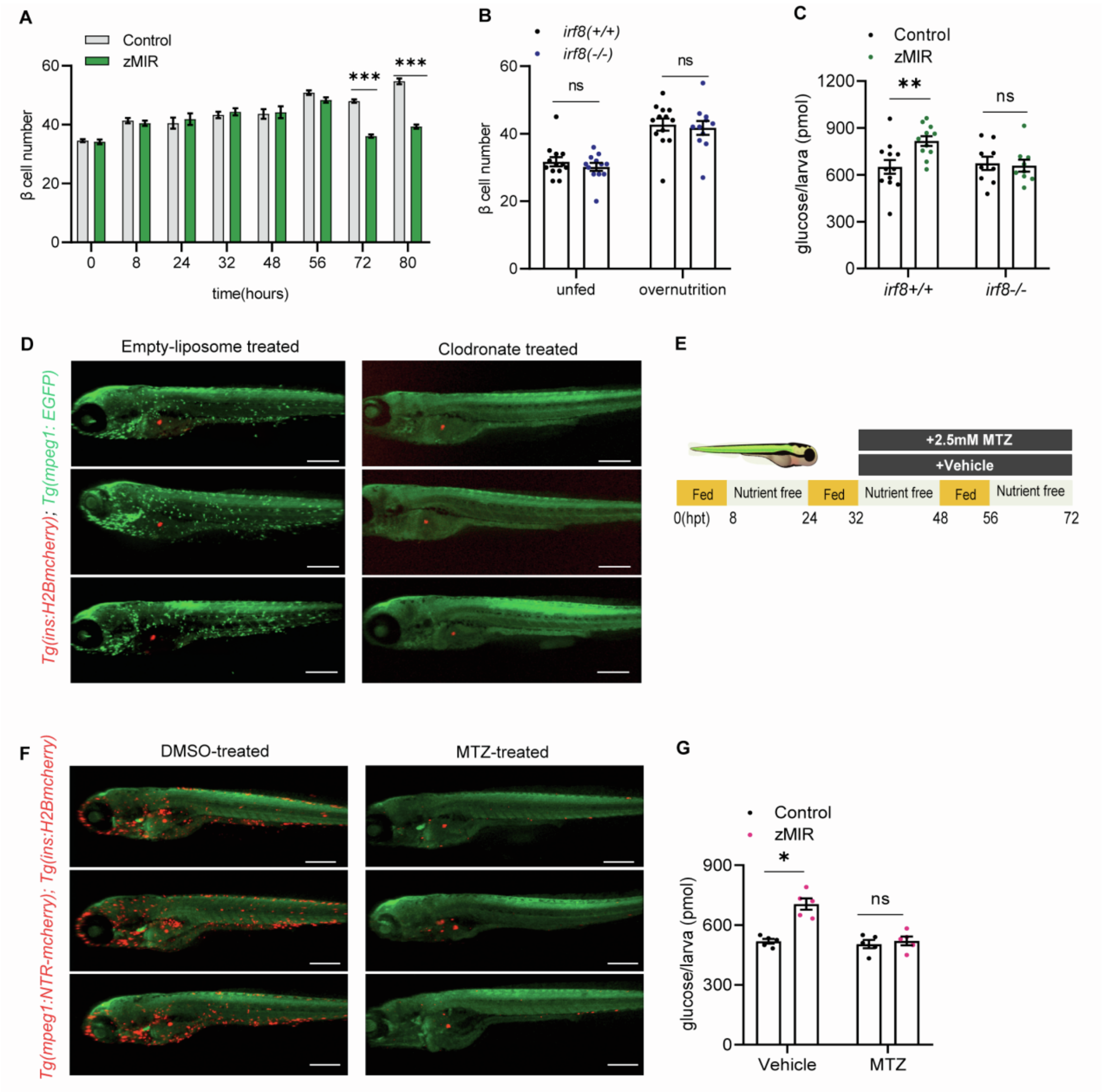

Figure S1. Macrophage is necessary for β cell loss. Related to Figure 1.

(A) β cell number in control and zMIR fish before 56 hpt. Unpaired t test, bar graphs represent mean ± SEM, n>30/group *** $p<0.001$.

(B) β cell number at 6 dpf with or without a single session of overnutrition in *irf8-/-* and control siblings. Unpaired t test, bar graphs represent mean ± SEM, n>10/group, ns, not significant.

(C) Effect of genetic deficiency of macrophage (*irf8-/-*) on whole-body free glucose content. Unpaired t test, bar graphs represent mean ± SEM, n>8/group, ns, not significant, ** $p<0.01$.

(D) Three representative images of *Tg(mpeg1:EGFP)* zebrafish injected with control empty-liposomes or with clodronate-liposomes (macrophages, green dots). Scale bars are 300 μm.

(E) Schematic for NTR-MTZ mediated macrophage ablation protocol.

(F) Three representative images of *Tg(mpeg1:NTR-mcherry)* zebrafish treated with DMSO or

or MTZ (macrophages, red dots). Scale bars are 300 μm.

(G) Effect of MTZ or vehicle on whole-body free glucose content in control and zMIR fish with *Tg(mpeg1:NTR-mcherry)* (n=10/group, 5 group for each treatment). Unpaired t test, bar graphs represent mean ± SEM, ns, not significant, * $p<0.05$.

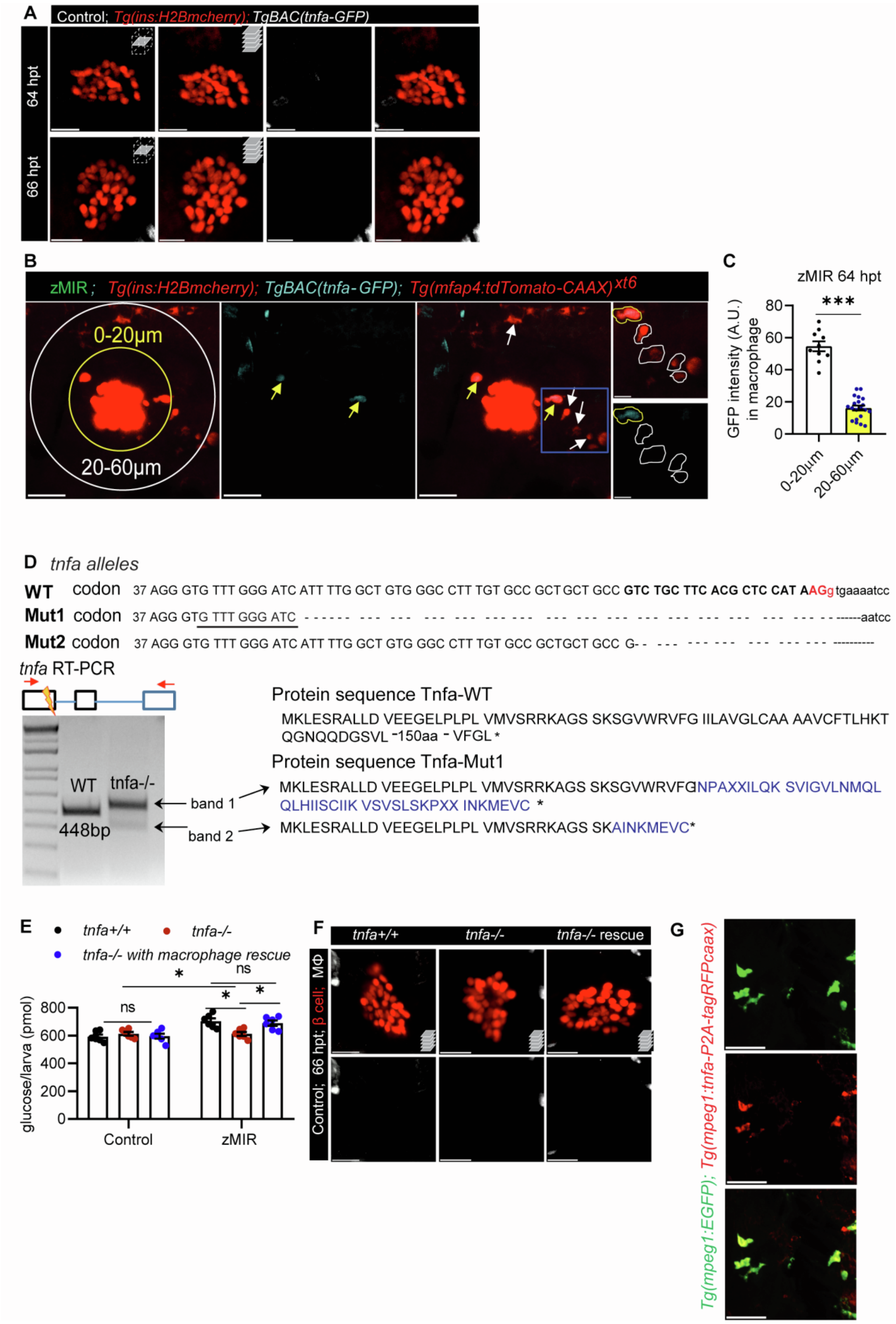

Figure S2. Macrophage-derived Tnfa is necessary for β cell loss in zMIR fish. Related to Figure 2.

(A) Islet images of *TgBAC(tnfa:GFP)*, *Tg(ins:H2b-mCherry)*, control fish at 64 and 66 hpt. GFP

signal was detected by immunofluorescence. Scale bars are 20 μm.

(B) Representative images showing higher EGFP signal in peri-islet macrophages in zMIR fish at 64 hpt. The fluorescent signals were from the native proteins without immunostaining. Scale bars are 50 μm.

(C) Quantification of GFP intensity from macrophages that are within 20 μm and within 20-60 μm of islet in zMIR fish at 64 hpt. Unpaired t test, bar graphs represent mean ± SEM, n=10/group, ns, not significant.

(D) Sequence comparison of wild-type and two mutant *tnfa* alleles and proteins. Top panel shows DNA sequence flanking the target site. Exonic sequence in capital, intronic sequence in lower case, target sequence in bold, PAM in red, and deletion as "–". The sequence that are aberrantly spliced out is underlined. All alleles result in frameshifts. Bottom left shows a schematic of the *tnfa* gene structure with rectangles as exons and lines as introns, the position of sgRNA target (lightning bolt), and positions for RT-PCR primers (red arrows), and a gel image showing RT-PCR results of WT and *tnfa*-/-, and the amplicon size of the WT product. A minor smaller product is present in the mutant cDNAs due to activation of a cryptic splice donor 10 bp upstream of the deletion. Bottom right shows the N-terminal 70 residues of WT Tnfa protein, the entire Mut1 protein and the predicted protein from the minor mRNA. Mut1 only contains 40 N-terminal residues of WT sequence followed by 47 residues of non-specific sequence (blue letters). The product from the aberrant splicing is predicted to contains only 38 N-terminal residues of WT sequence followed by 10 gibberish residues (blue letters).

(E) Re-expression of *tnfa* in macrophage is sufficient to increase the whole-body free glucose content in zMIR fish after three sessions of overnutrition (n=10/group, 6 group for each treatment). Data are mean ± SEM, multiple t test, ns, not significant, * $p<0.05$.

(F) Representative images showing intra-islet macrophages in control, *tnfa-/-*, and *tnfa-/-* with *tnfa* re-expression in macrophages at 66 hpt. Scale bars are 10 μm.

(G) Confirmation of macrophage specific *tnfa* expression in *Tg(mpeg1:EGFP)*; *Tg(mpeg1:tnfa-P2A-tagRFPcaax)* fish at 5 dpf (before overnutrition). Scale bars are 20 μm.

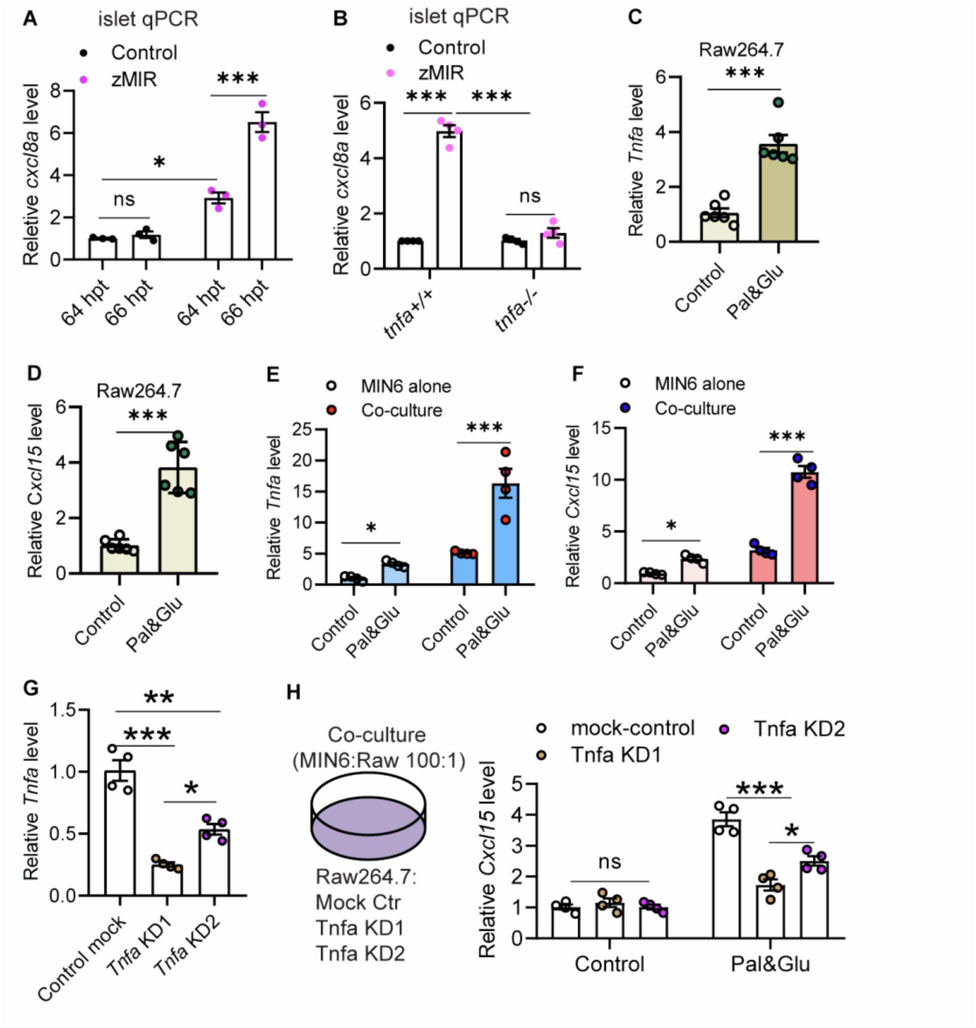

Figure S3. A conserved TNFA-*CXCL8* axis in β cells under ER stress. Related to Figure 3.

(A) qRT-PCR analysis of islet *cxcl8a* expression at 64 and 66 hpt in both control and zMIR fish. Data are mean ± SEM, n = 3/group, two-way ANOVA, followed by Tukey's multiple comparisons test, ns, not significant, * p<0.05, *** p<0.001.

(B) qRT-PCR analysis of islet *cxcl8a* expression at 66 hpt in *tnfa-/-*, zMIR and control zMIR fish. Data are mean ± SEM, n = 3/group, two-way ANOVA, followed by Tukey's multiple comparisons test, ns, not significant, *** p<0.001.

(C-D) *Tnfa* and *Cxcl15* expression in Raw264.7 cells culture in normal or Pal&glu medium. Unpaired t test, bar graphs represent mean ± SEM, n=6/group, *** p<0.001.

(E-F) *Tnfa* and *Cxcl15* expression in MIN6 alone with co-cultured with Raw264.7 cell (100:1). Data are mean ± SEM, n = 4/group, Multiple t test, * p<0.05, *** p<0.001.

(G) Knockdown efficiencies of *Tnfa* in raw264.7 by two Cas13d sgRNAs. Data are mean ± SEM, n = 4/group, one-way ANOVA, followed by Tukey's multiple comparisons test, ns, not significant, * p<0.05, ** p<0.01, *** p<0.001.

(H) *Cxcl15* expression in MIN6 cells cultured in normal (control) or pal&glu conditioned

medium from the *Tnfa* knockdown Raw264.7 cells. Data are mean ± SEM, multiple t test, n=4/group, ns, not significant, * $p<0.05$, *** $p<0.001$.

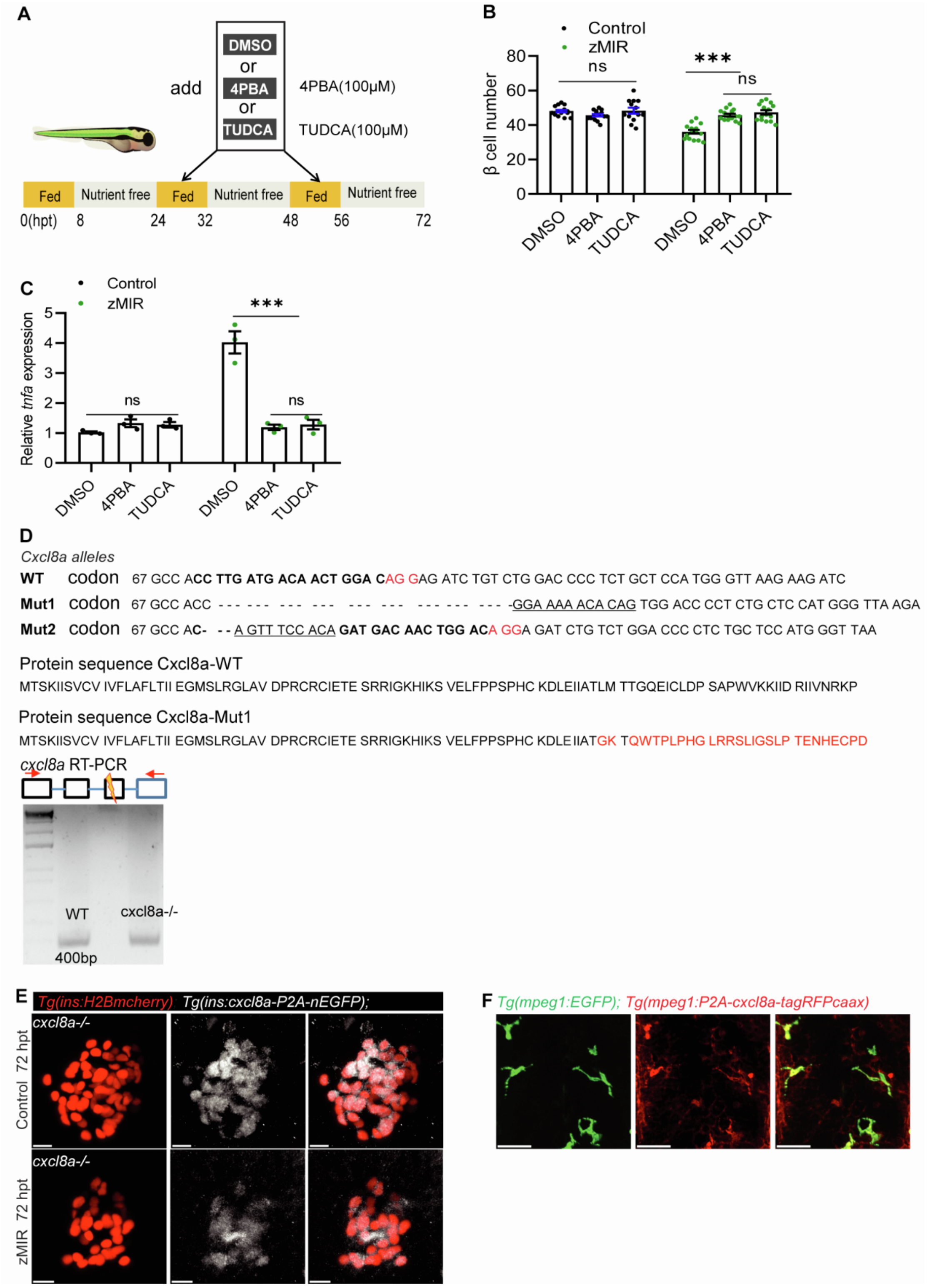

Figure S4. β cell-derived Cxcl8a is required for β cell loss in zMIR fish. Related to Figure 4.

(A) Schematic showing experimental protocol for administration of 4PBA and TUDCA. Compounds were added in second and third overnutrition sessions.

(B) Effect of ER stress inhibitors (TUDCA or 4PBA) on β-cell loss. Data represent mean ± SEM,

n > 10/group, two-way ANOVA, followed by Tukey's multiple comparisons test, ns, not significant, *** $p<0.001$.

(C) Islet qRT-PCR analysis of *tnfa* at 66 hpt in control and zMIR fish treated with DMSO (Vehicle), TUDCA or 4PBA at 66 hpt. Data are mean ± SEM, n = 3/group, multiple t test, followed by Tukey's multiple comparisons test, ns, not significant, *** $p<0.001$.

(D) Sequence comparison of WT and mutant *cxcl8a* alleles and proteins. Top panel shows DNA sequence flanking the target site. Exonic sequence is in capital, intronic sequence in lower case, target sequence in bold, PAM in red, insertion underlined, and deletion as "–". Both alleles result in frameshift. Middle panel shows the entire sequence of WT Cxcl8a protein and predicted Mut1 protein, indicating the deletion of C-terminal 29 residues in the mutant. Bottom panel schematic shows cxcl8a gene structure with rectangles as exons and lines as introns, sgRNA target site (lightning bolt), and primer positions (red arrows). The gel image shows RT-PCR product of WT and *cxcl8a-/-*. No additional product is present in the mutant cDNA.

(E) Confirmation of β-cell specific *cxcl8a* expression in *Tg(ins:H2BmCherry)*; *Tg(ins:cxcl8a-P2A-nEGFP)* zMIR fish at 72 hpt (after 3 overnutrition). Scale bars are 10 μm.

(F) Confirmation of macrophage specific *cxcl8a* expression in *Tg(mpeg1:EGFP); Tg(mpeg1:cxcl8a-P2A-tagRFP$_{caax}$)* fish at 5 dpf (before overnutrition). Scale bars are 10 μm.

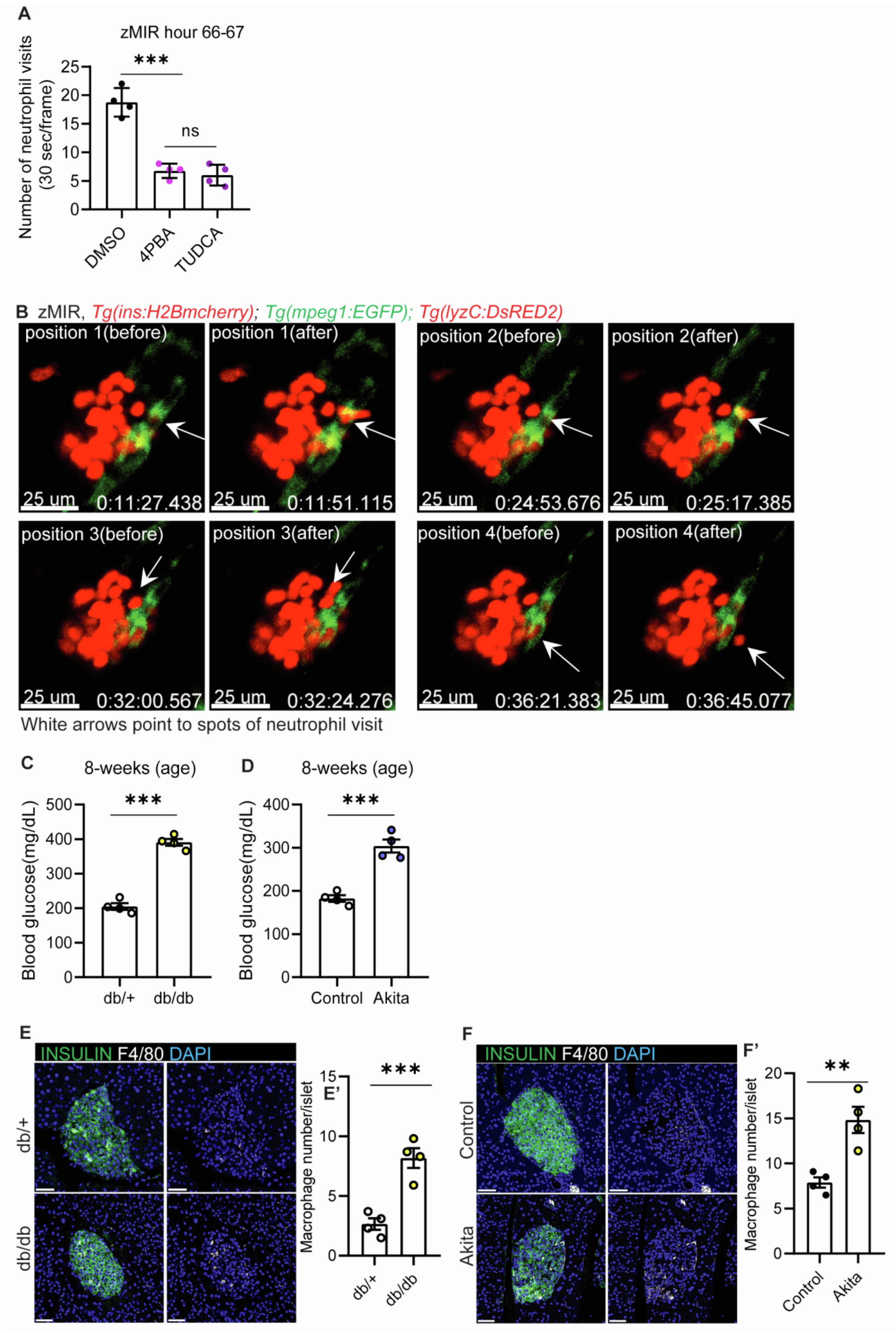

Figure S5. Cxcl8a is necessary for recruiting neutrophils to the islet. Related to Figure 5.

(A) Quantification of islet visits by neutrophils in live image videos from 66 to 67 hpt of zMIR fish treated with DMSO, 4PBA or TUDCA. Videos were recorded at 30s intervals. Data are

mean ± SEM, n = 4/group, one-way ANOVA, followed by Tukey’s multiple comparisons test, ns, not significant, *** p<0.001.

(B) Selected frames from a representative video of zMIR fish from 66 to 67 hpt showing neutrophils and macrophages in the islet. Videos were recorded at 30s intervals. The macrophage was labeled with GFP, neutrophils were labeled with DsRed, β cells were labeled with mCherry. White arrows point to a position where macrophages (green cells) and a neutrophil (red cells) sequentially occupied. (Related to supplemental video 8).

(C) Random blood glucose levels of 8-week-old *db/+* (B6.BKS(D)-Leprdb/J) and *db/db* mice. Unpaired t test, bar graphs represent mean ± SEM, n=4/group, *** p<0.001

(D) Random blood glucose levels of 8-week-old control (C57BL/6) and Akita (C57BL/6-Ins2Akita/J) mice. Unpaired t test, bar graphs represent mean ± SEM, n=4/group, *** p<0.001

(E) Representative immunofluorescence images of F4/80 and insulin in pancreas sections of 8-week-old *db/+* (B6.BKS(D)-Leprdb/J) and *db/db* mice, Scale bars are 50μm.

(E’) Quantification of intra-islet macrophage number in *db/+* and *db/db* mice. (n=4, at least 50 islets were quantified in each animal). Unpaired t test, bar graphs represent mean ± SEM, *** p<0.001.

(F) Representative immunofluorescence images of F4/80 and inuslin in pancreas sections of 8-week-old control (C57BL/6) and Akita (C57BL/6-Ins2Akita/J) mice, Scale bars are 50μm.

(F’) Quantification of intra-islet macrophage number in control and Akita mice. (n=4, at least 50 islets were quantified in each animal). Unpaired t test, bar graphs represent mean ± SEM, *** p<0.001.

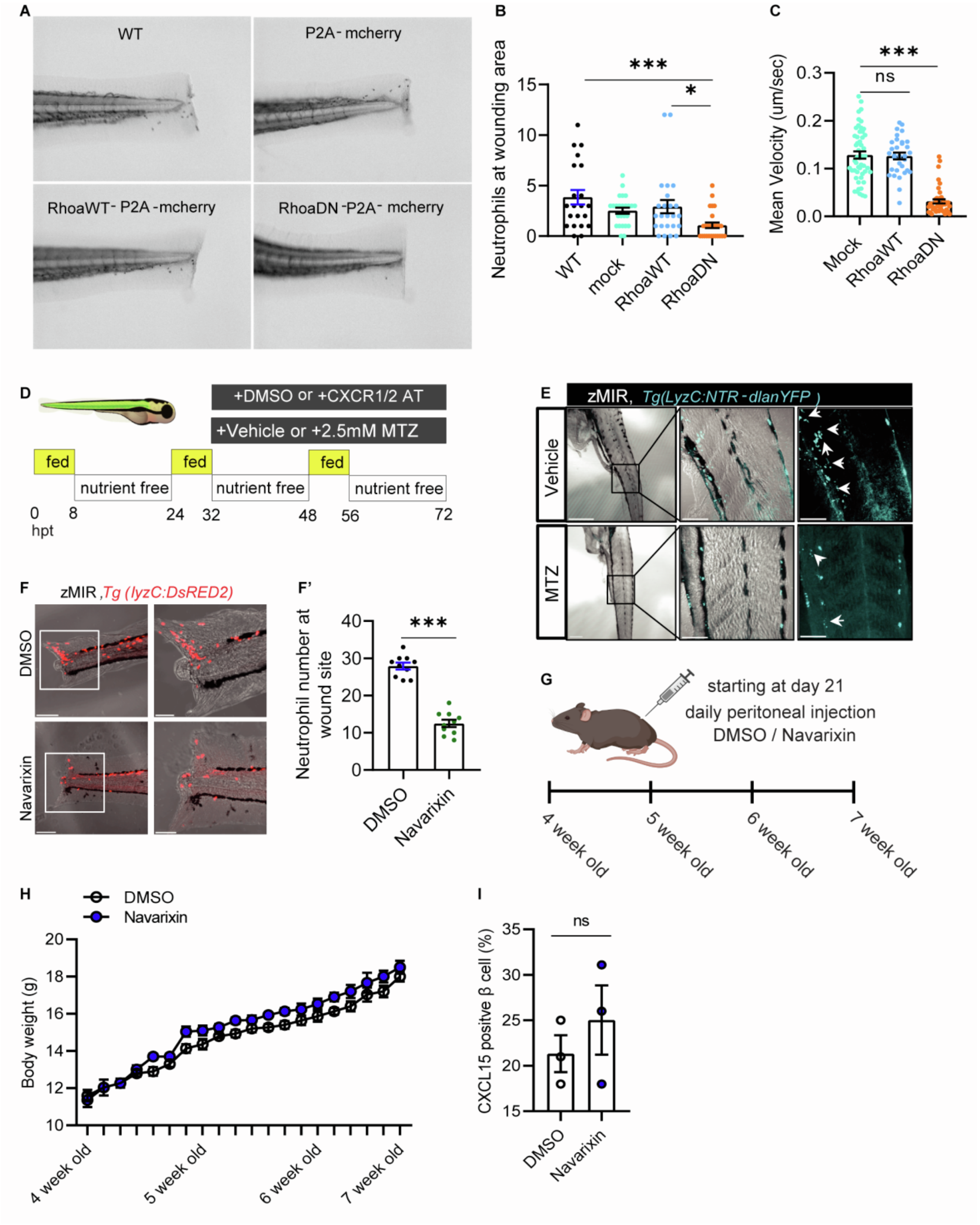

Figure S6. Islet neutrophils are necessary for β cell loss. Related to Figure 6.

(A-B) Effect of $Rhoa^{DN}$ expression by *Tg(LyzC:mCherry-2a-Rhoa^{DN})* on neutrophil recruitment at amputation site. Data are mean ± SEM, n = 25 per group, one-way ANOVA, followed by Tukey’s multiple comparisons test, * p<0.05, *** p<0.001.

(C) Effect of $Rhoa^{DN}$ expression on neutrophil mean velocity. Data are mean ± SEM, n > 30 per group, one-way ANOVA, followed by Tukey’s multiple comparisons test, ns, not significant, *** p<0.001.

(D) Schematic showing experimental protocol for administration of Cxcr1/2 antagonist and

MTZ.

(E) Representative images of neutrophils (cyan) in *Tg(LyzC:NTR-dlanYFP)* fish treated with vehicle or MTZ. Scale bars are 50 μm.

(F) Representative images showing the effect of Navarixin on neutrophil recruitment to the amputation site. Scale bars are 50 μm.

(F’) Quantification of neutrophil recruitment to the amputation site in fish treated with vehicle or Navarixin (n=10 per group). Unpaired t test, bar graphs represent mean ± SEM, *** p<0.001.

(G) Schematic showing experimental protocol for daily peritoneal injection with DMSO or Navarixin (5μg/g) in *Akita* (*C57BL/6-Ins2Akita/J*) mice, starting at 3-week-old (day 21) (n=3).

(H) Body weight of *Akita* (*C57BL/6-Ins2Akita/J*) mice injected with DMSO or Navarixin. Multiple t test, n=3, graphs represent mean ± SEM.

(I) Quantification of the percentage of CXCL15 positive β cells in *Akita* (*C57BL/6-Ins2Akita/J*) mice injected with DMSO or Navarixin (n=3, at least 50 islets were quantified in each animal). Unpaired t test, bar graphs represent mean ± SEM, ns, not significant.

Table S1. qPCR primers used in this study. Related to Figure 3 and Figure S3

zebrafish	Forward	Reverse
eifa	CTTCTCAGGCTGACTGTGC	CCGCTAGCATTACCCTCC
cxcl8a	TGTGTTATTGTTTTCCTGGCATTTC	GCGACAGCGTGGATCTACAG
tnfa	AGACCTTAGACTGGAGAGATGAC	CAAAGACACCTGGCTGGCTGTAGAC
Mouse		
gapdh	CCAGCTACTCGCGGCTTTA	GAGGGCTGCAGTCCGTATTT
il1b	TGCCACCTTTTGACAGTGATG	AAGGTCCACGGGAAAGACAC
Tnfa	TCAAACCCTGGTATGAGCCC	ACCCATTCCCTTCACAGAGC
cxcl15	AGGAAGTGATAGCAGTCCCAA	CAGAAGCTTCATTGCCGGTG
Human		
cxcl8	CCACCCCAAATTTATCAAAGAA	CAGACAGAGCTCTCTTCCATCA
gapdh	CAATGCCTCCTGCACCACCA	GATGTTCTGGAGAGCCCCGC
